# Supplementary material for: Ageing and brain white matter structure in 3,513 UK Biobank participants
Source: Nat Commun. 2016 Dec 15;7:13629. doi: 10.1038/ncomms13629 (PMC5172385; doi:10.1038/ncomms13629)
Supplement: Supplementary Information — Supplementary Figures 1 - 16 and Supplementary Tables 1 - 20 [file ncomms13629-s1.pdf]

Supplementary Figures

Supplementary Figure 1. Flowchart of scanning and quality control for initial release of UK Biobank tract-averaged DT-MRI data.

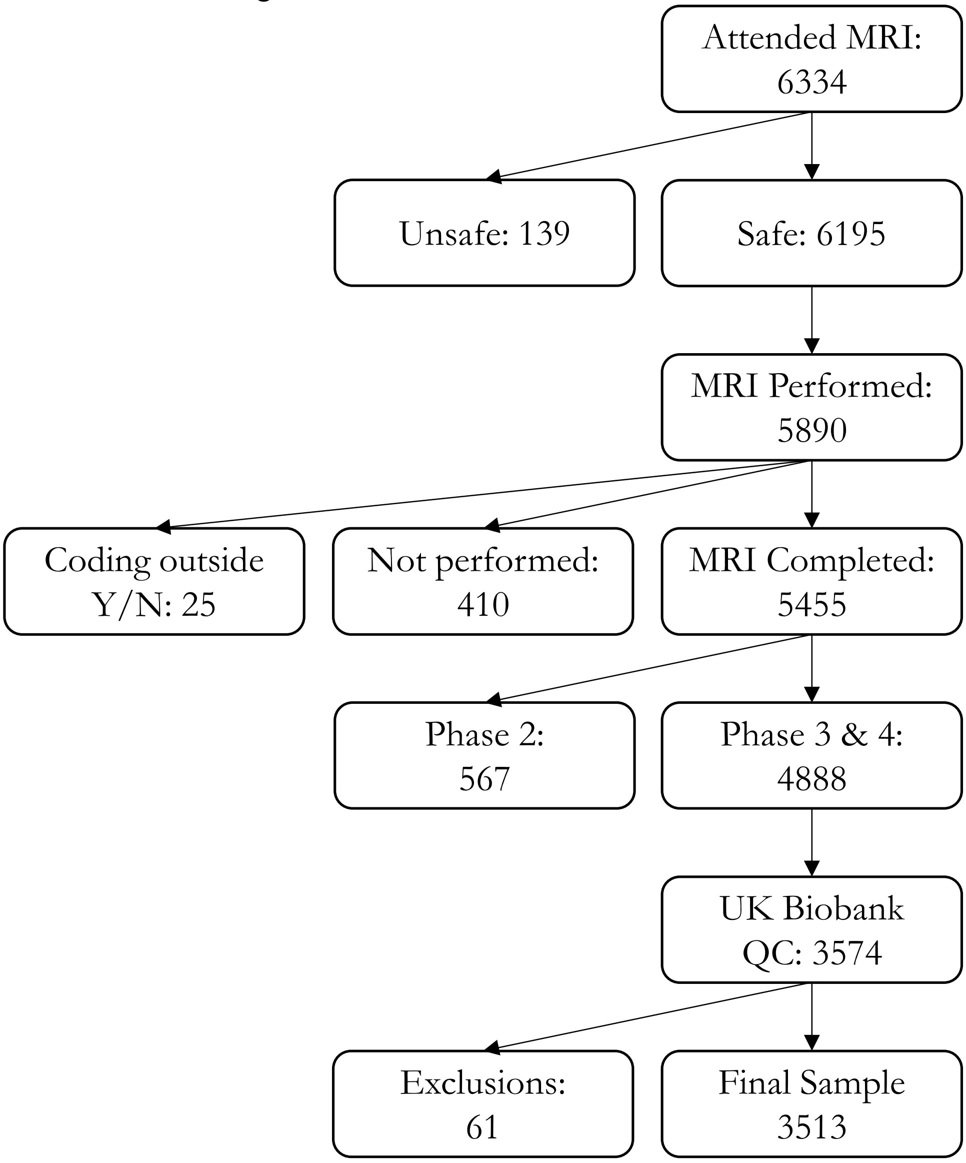

Note. Following safety ascertainment, 5,455 MRIs were completed. Of these, 4,888 comprised compatible DT-MRI data, and a total of 3,513 passed quality control (QC) procedures and exclusion criteria. Coding outside refers to instances in which yes or no were not applicable (see http://biobank.ctsu.ox.ac.uk/crystal/field.cgi?id=12188). DT-MRI acquired in Phase 2 was not comparable to subsequent phases, and was therefore removed from analysis.

Supplementary Figure 2. Tract-averaged diffusion characteristics of brain white matter in UK BioBank.

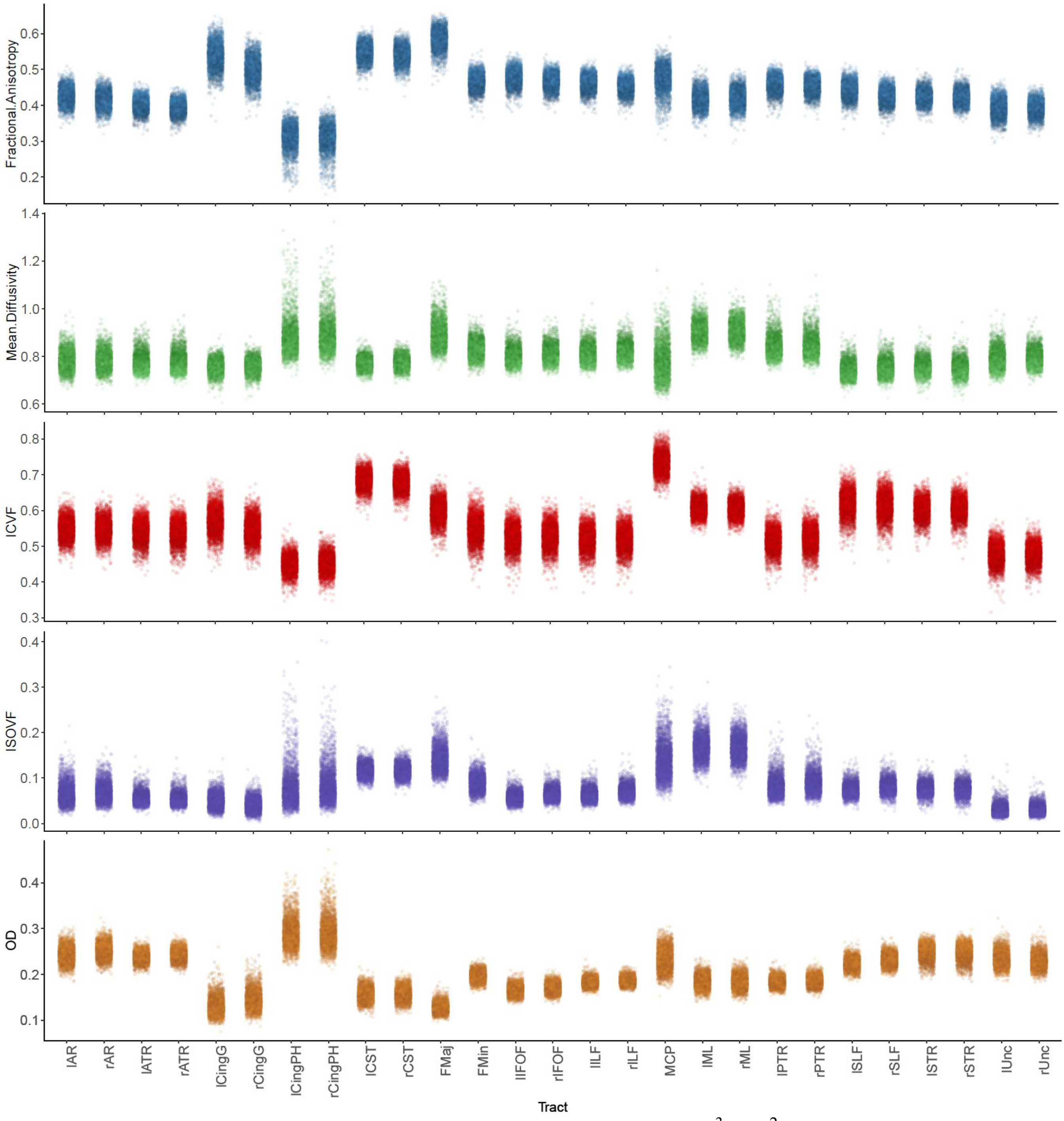

Note. FA = fractional anisotropy, MD = Mean diffusivity ($\times10^{-3}$ mm^2/s), ICVF = intracellular volume fraction, ISOVF = isotropic volume fraction, OD = orientation dispersion. AR: acoustic radiation, ATR: anterior thalamic radiation, CingG: cingulum gyrus, CingPH: cingulum parahippocampal, CST: corticospinal tract, FMaj: forceps major, FMin: forceps minor, IFOF: inferior fronto-occipital fasciculus, ILF: inferior longitudinal fasciculus, MCP: middle cerebellar peduncle, ML: medial lemniscus, PTR: posterior thalamic radiation, SLF: superior longitudinal fasciculus, STR: superior thalamic radiation, Unc: uncinate fasciculus, l: left, r: right. Data has been jittered for visualisation purposes.

Supplementary Figure 3. Tract-averaged characteristics of supplementary brain white matter parameters in UK BioBank.

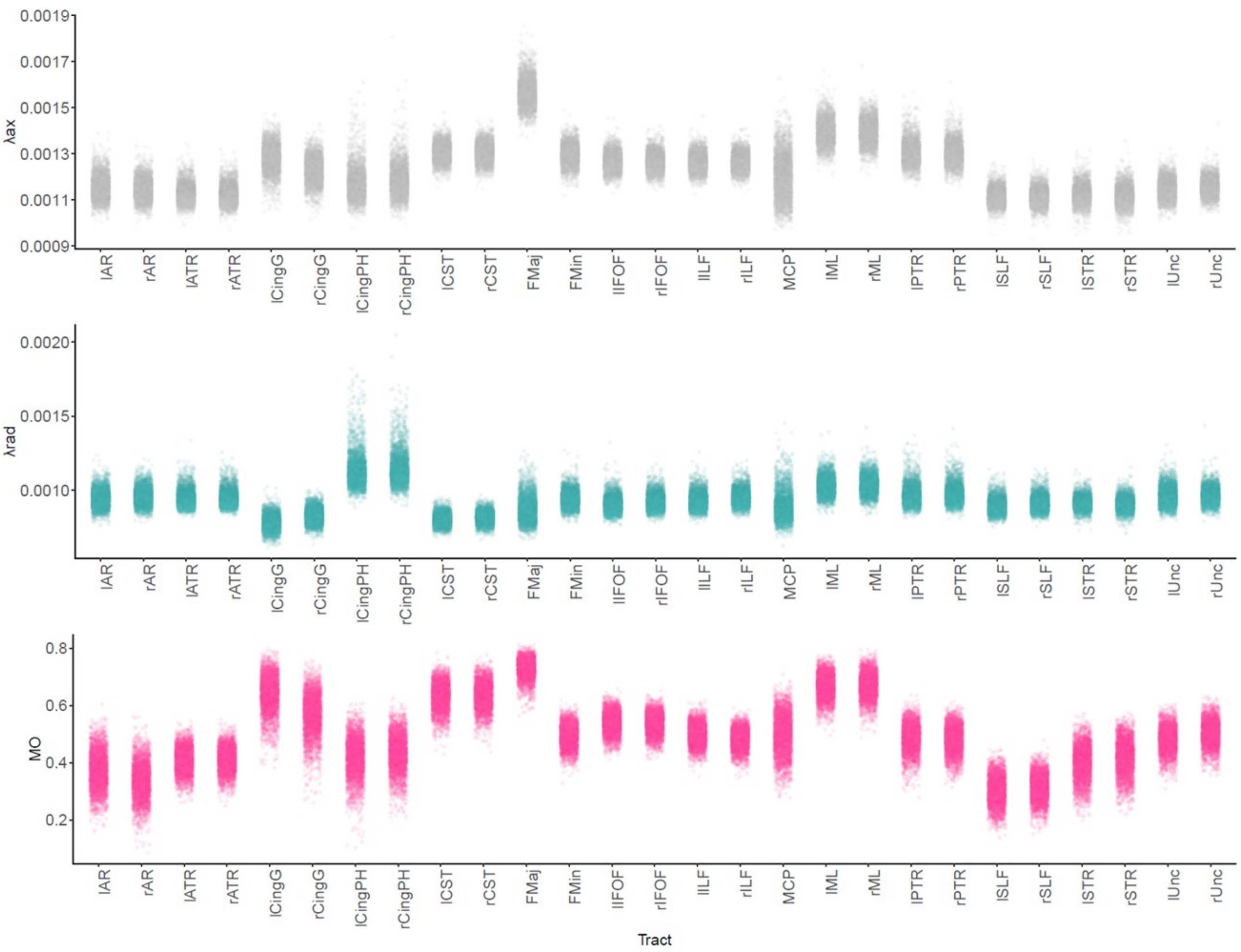

Note. λax = axial diffusivity, λrad = radial diffusivity, MO = diffusion tensor mode. AR: acoustic radiation, ATR: anterior thalamic radiation, CingG: cingulum gyrus, CingPH: cingulum parahippocampal, CST: corticospinal tract, FMaj: forceps major, FMin: forceps minor, IFOF: inferior fronto-occipital fasciculus, ILF: inferior longitudinal fasciculus, MCP: middle cerebellar peduncle, ML: medial lemniscus, PTR: posterior thalamic radiation, SLF: superior longitudinal fasciculus, STR: superior thalamic radiation, Unc: uncinate fasciculus, l: left, r: right. Data has been jittered for visualisation purposes.

Supplementary Figure 4. Heatmaps of inter- and intra-hemispheric associations (Pearson's r) for tract fractional anisotropy (blue), mean diffusivity (green), ICVF (red), ISOVF (purple), OD (orange), MO (pink and blue), λax (grey) and λrad (turquoise).

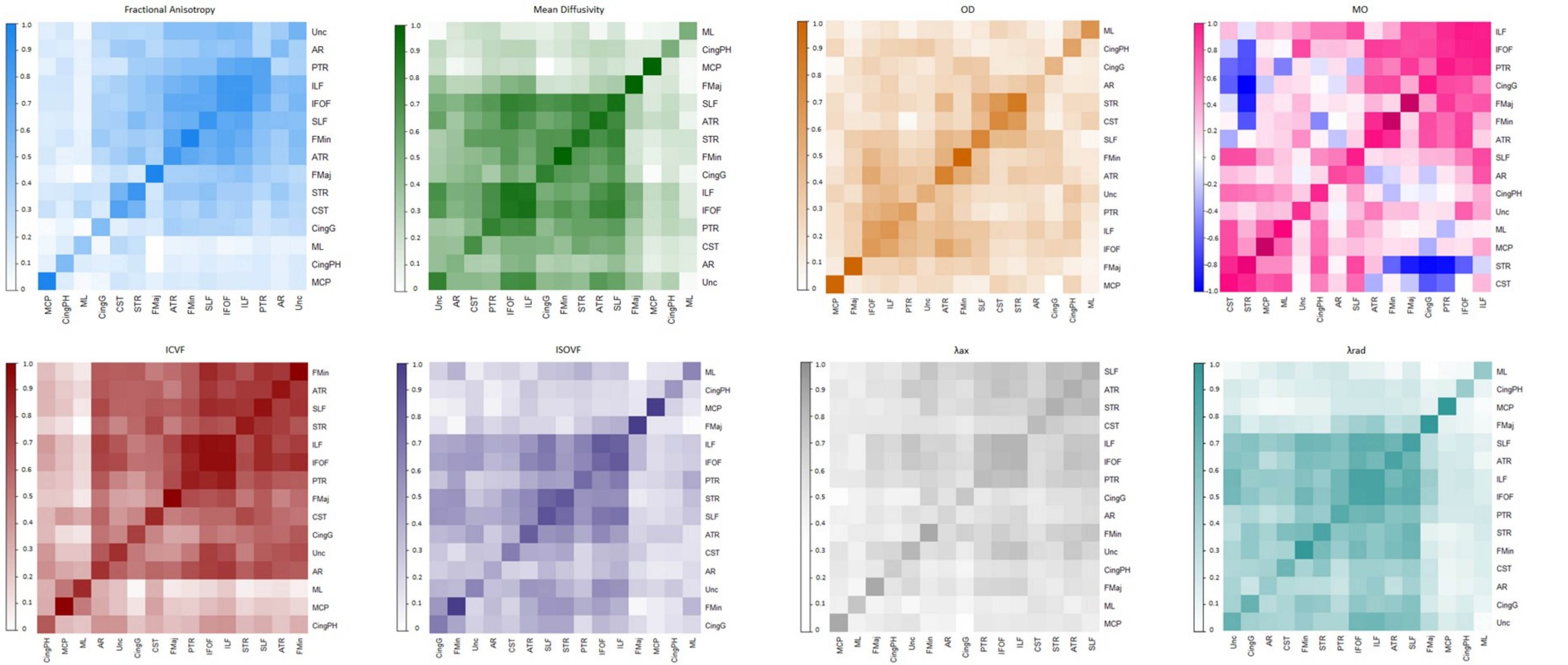

Note. Y-axis denotes left hemisphere tracts, central diagonal indicates left:right associations of tracts, except for the MCP, FMaj and FMin (where r = 1). In each case, the heatmaps are arranged by grouping highly-correlated variables around the diagonal. The comparative absence of correlations among those tracts that show the lowest age effects (pale "L" shape; FMaj, MCP, CingPH and ML) is notable for FA, MD, λrad, ICVF and ISOVF.

Supplementary Figure 5. Heatmap of inter- and intra-hemispheric associations (Pearson's r) among FA, MD, IVCF, ISOVF and OD.

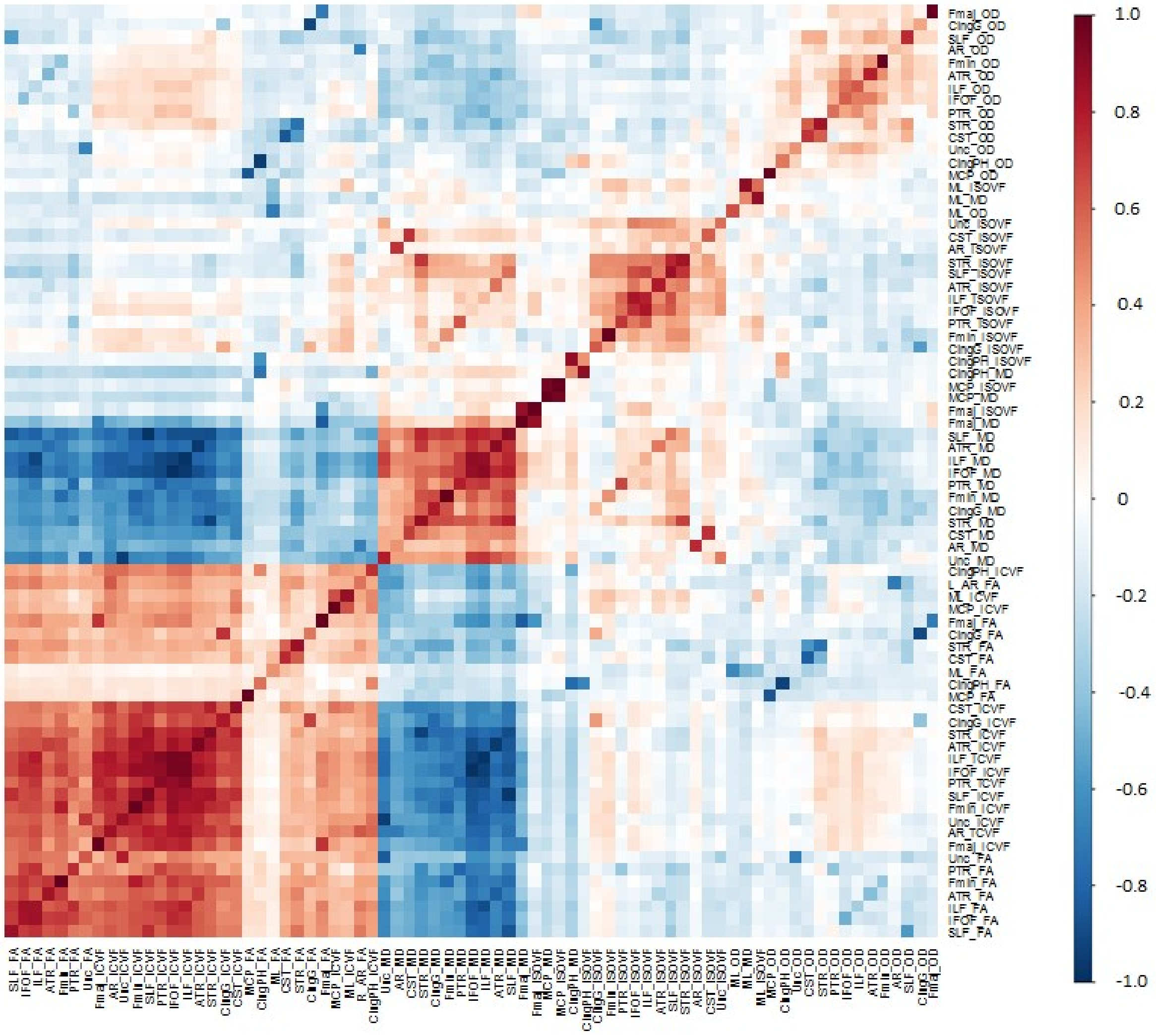

Note. Y-axis denotes left hemisphere tracts, central diagonal indicates left:right associations of tracts, except for the MCP, FMaj and FMin (where r = 1). Correlations are arranged by grouping highly-correlated variables around the diagonal.

Supplementary Figure 6. Left: Scree slopes for the exploratory factor analysis, showing the eigenvalue against the number of factors for λax, λrad and MO. Centre: Age trajectories of the first (latent) factor of white matter microstructure for λax and λrad. Right: Age de-differentiation of white matter microstructure.

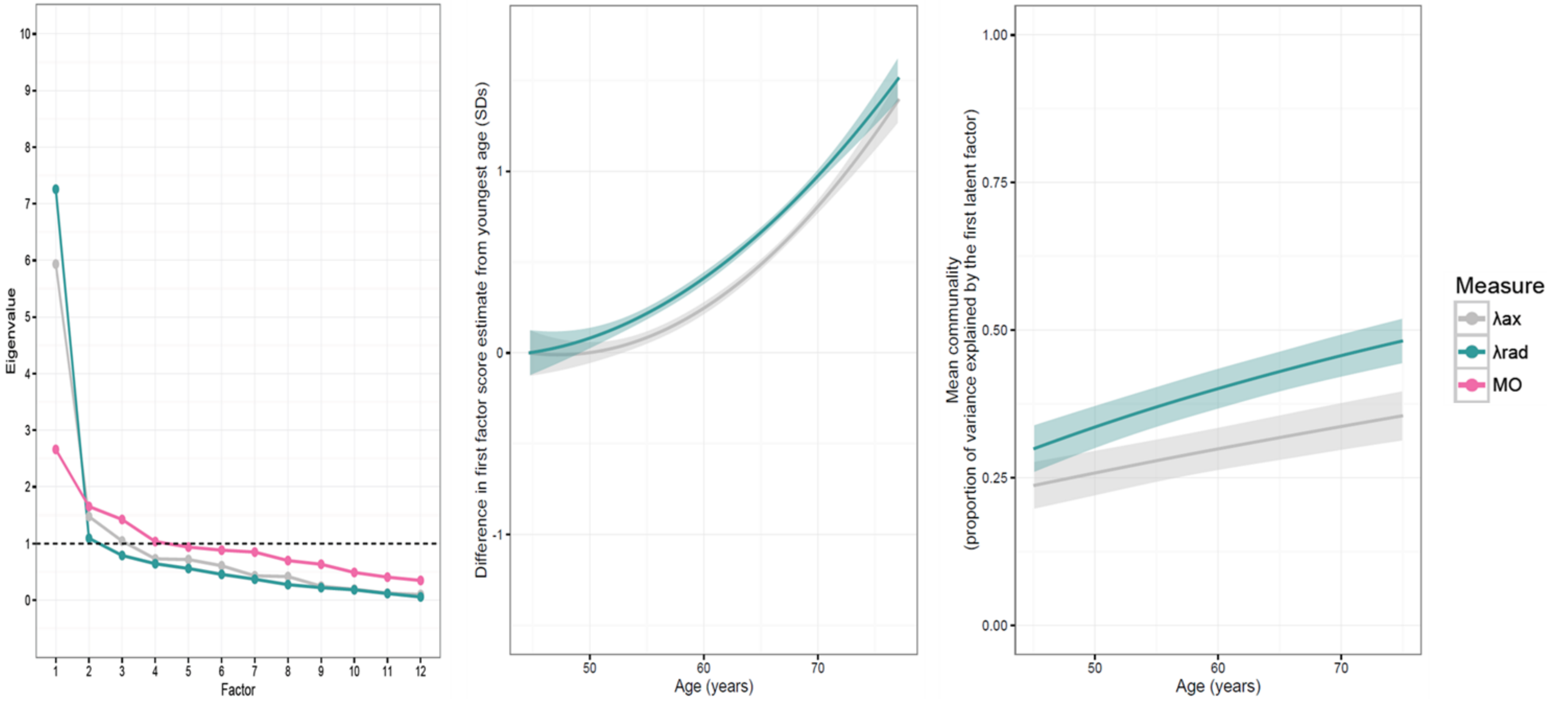

Note. Age trajectories for the proportion of total variance in each tract measurement explained by the general factor for λax and λrad. The shaded region around each trajectory shows ±1 SD of the mean.

Supplementary Figure 7. Common + independent pathways models for latent microstructural indices.

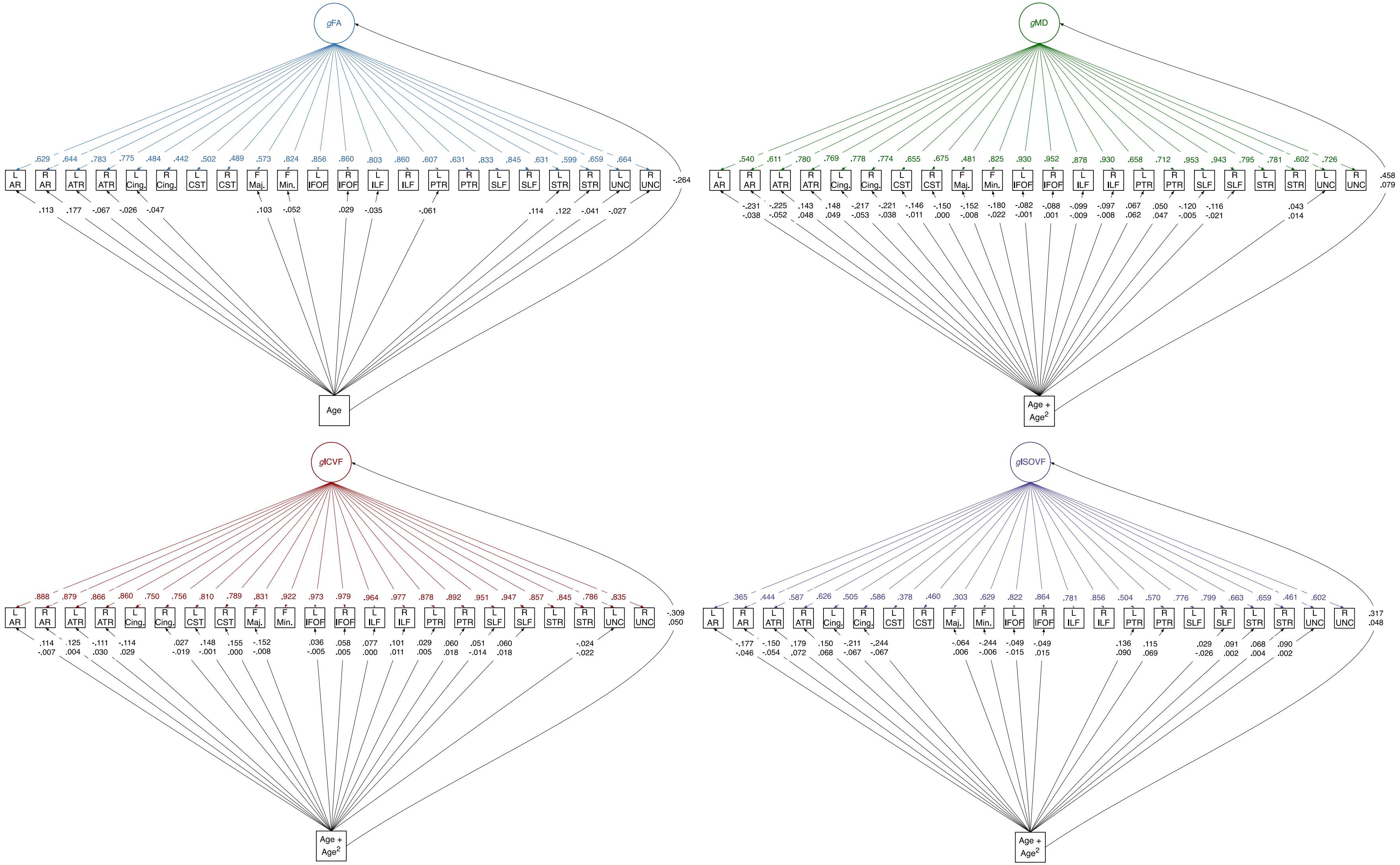

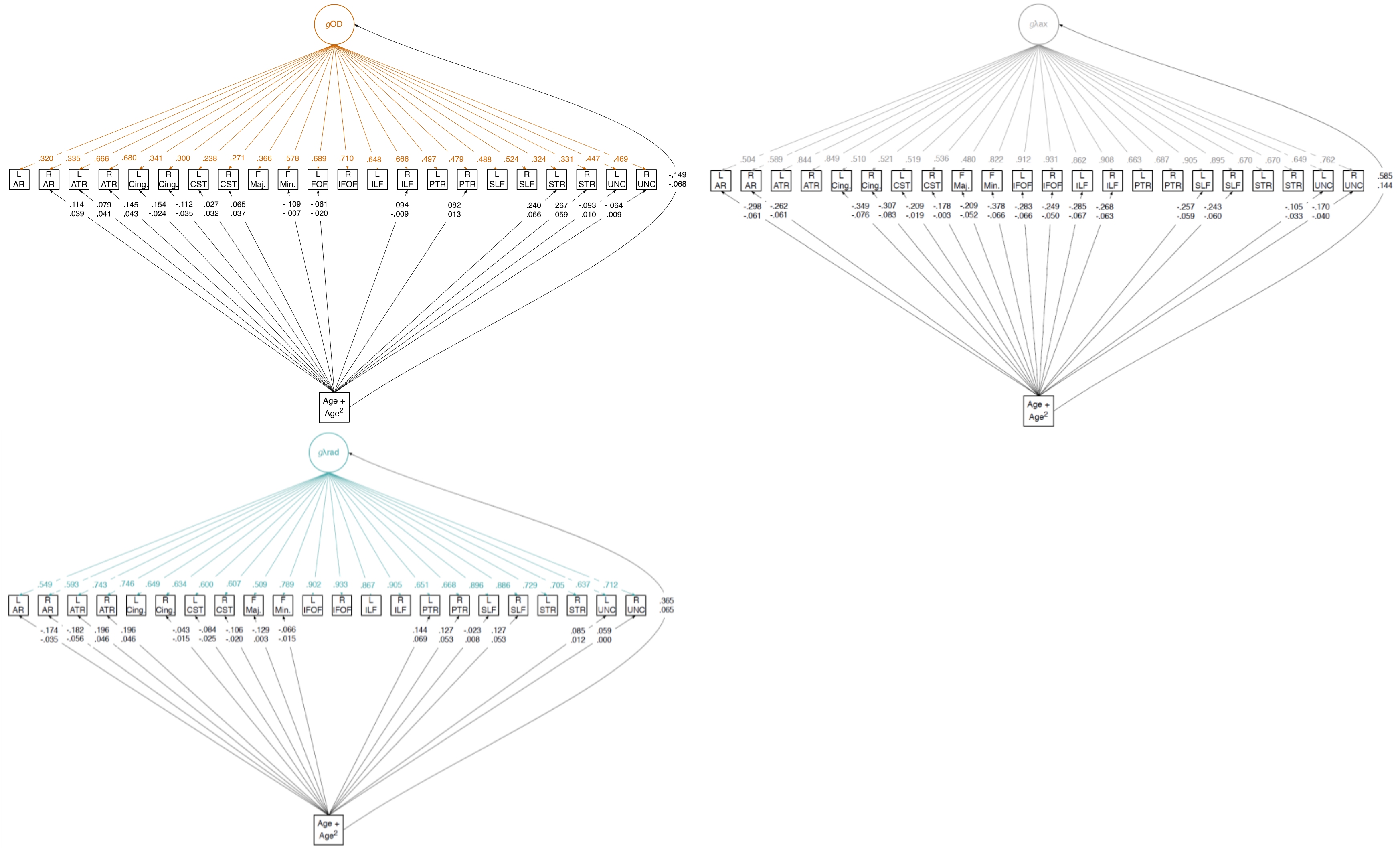

Note. Values are standardized path coefficients. For clarity, between-tract residual correlations are not shown here. FA (blue): fractional anisotropy, MD (green): mean diffusivity, ICVF (red): intracellular volume fraction, ISOVF (purple): isotropic volume fraction, OD (orange): orientation dispersion, grey: λax, turquoise: λrad. Squares indicate manifest variables and ellipses indicate latent variables.

Supplementary Figure 8. Mediation model for age, gFA, gICVF, and gOD.

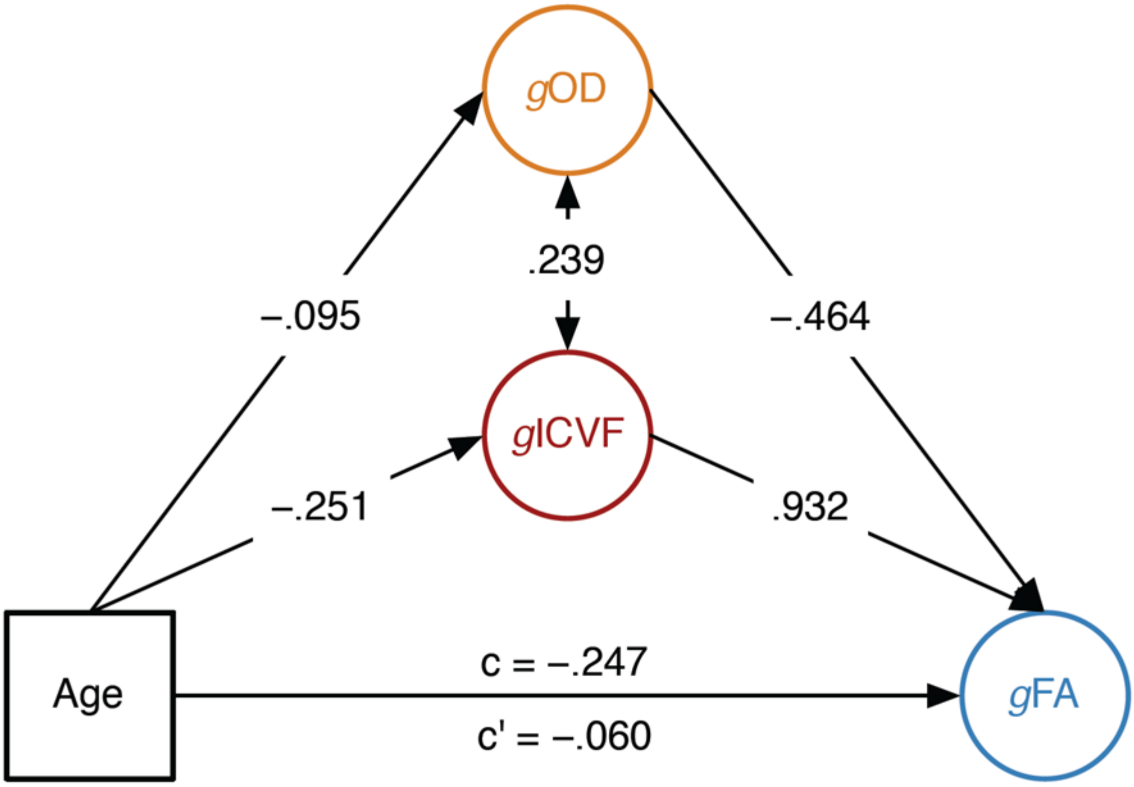

Note. The relationship between gFA and age (path c) is 75% mediated (note lower value of path c') via a combination of gICVF and gOD. The majority of the mediation takes place through gICVF rather than OD (respective indirect effects are β = -0.232 and β = 0.044; see Supplementary Table 8). Standardised βs reported. Squares indicate manifest variables and ellipses indicate latent variables.

Supplementary Figure 9. Associations between volumetric brain MRI measures and age.

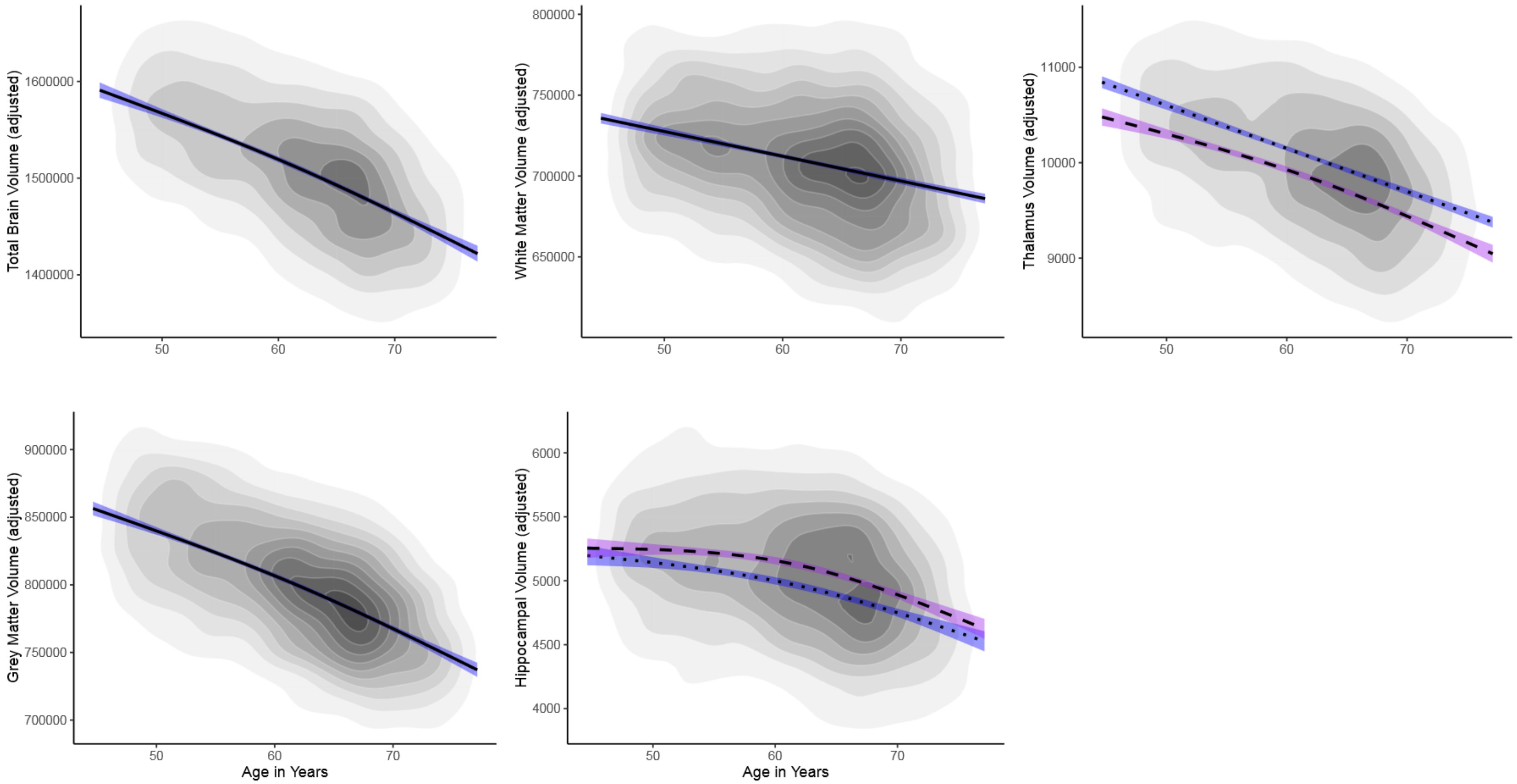

Note. Volumes in mm^3, adjusted for head size. Shaded area around the regression lines are 95% CIs. Dotted line denotes left hippocampus or thalamus, dashed line denotes right.

Supplementary Figure 10. De-differentiation trajectories for FA.

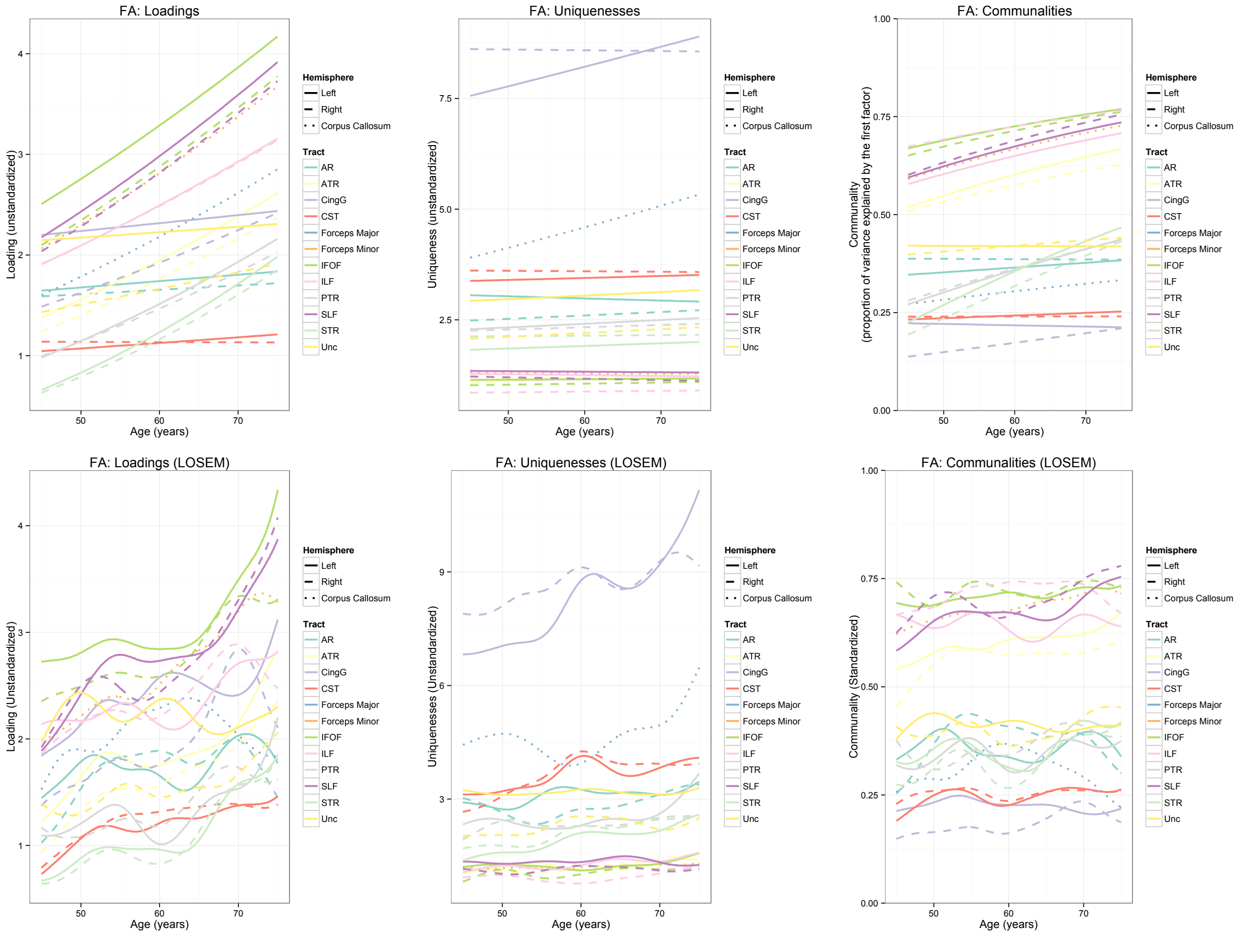

Note. Trajectories for factor loadings (left), tract uniquenesses (centre) and tract communalities (right) for fractional anisotropy (FA), taken from the multi-parameter age moderation model (top row) and LOSEM model (bottom row).

Supplementary Figure 11. De-differentiation trajectories for MD.

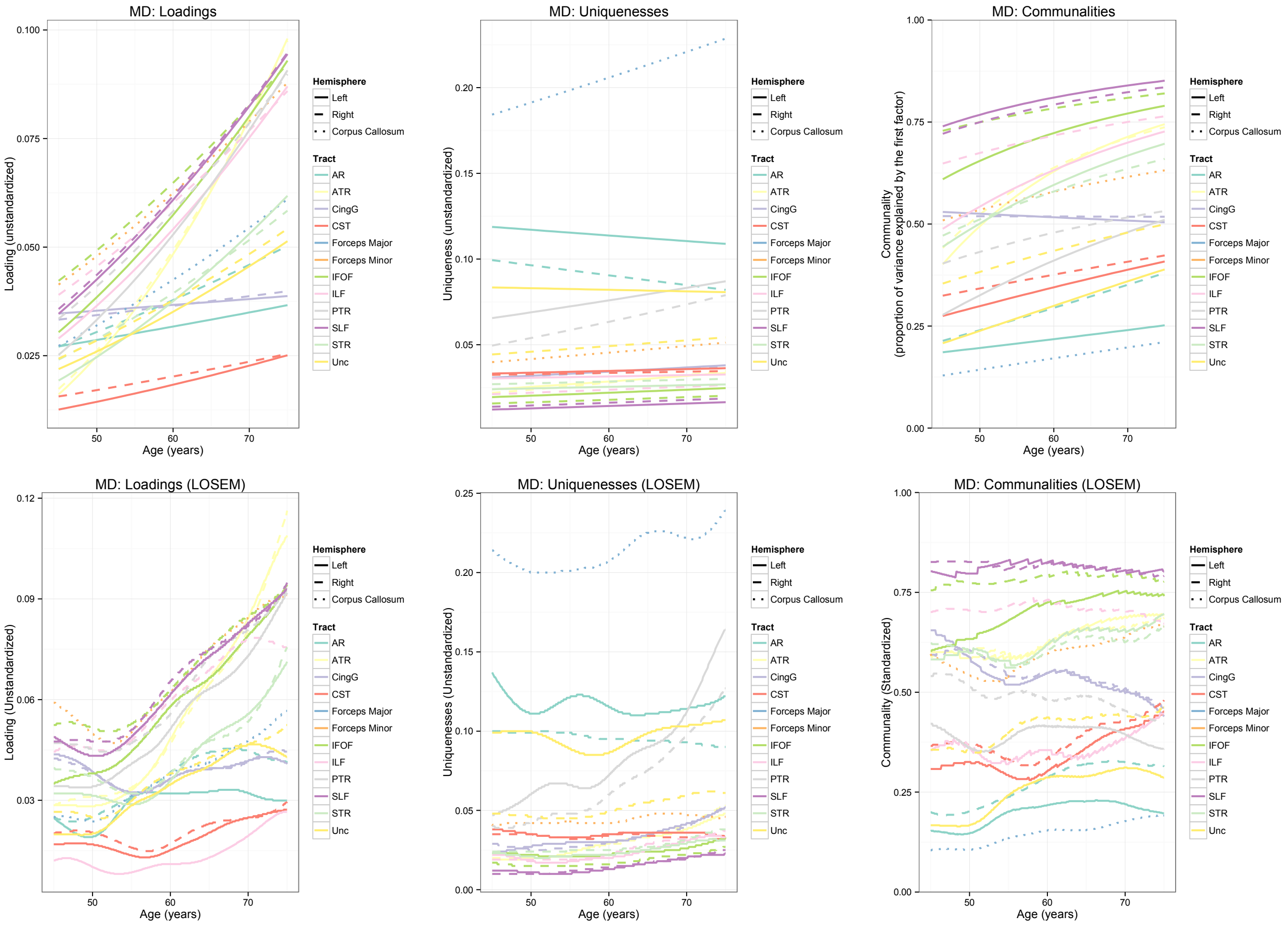

Note. Trajectories for factor loadings (left), tract uniquenesses (centre) and tract communalities (right) for mean diffusivity (MD), taken from the multi-parameter age moderation model (top row) and LOSEM model (bottom row).

Supplementary Figure 12. De-differentiation trajectories for ICVF.

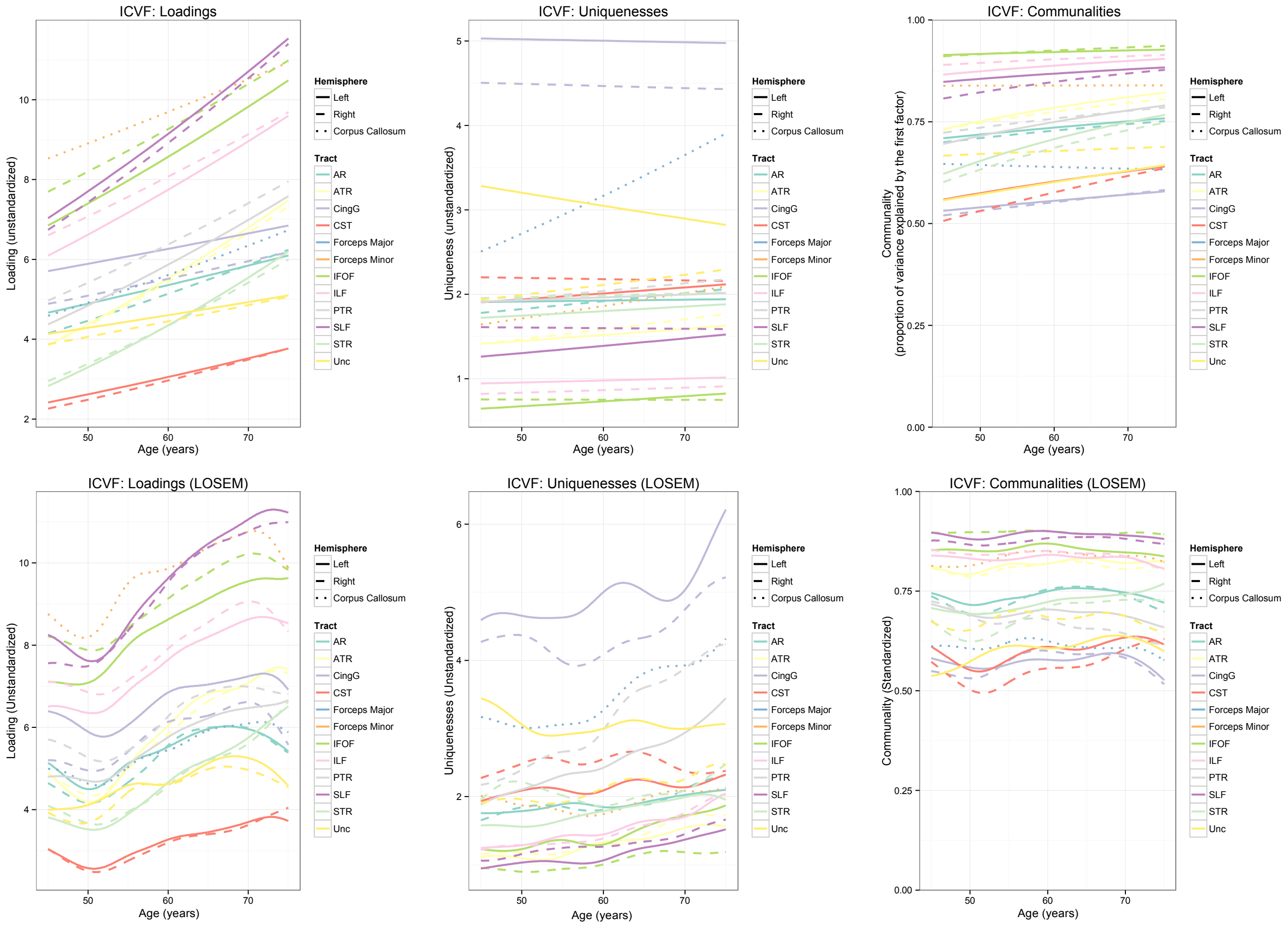

Note. Trajectories for factor loadings (left), tract uniquenesses (centre) and tract communalities (right) for intracellular volume fraction (ICVF), taken from the multi-parameter age moderation model (top row) and LOSEM model (bottom row).

Supplementary Figure 13. De-differentiation trajectories for ISOVF.

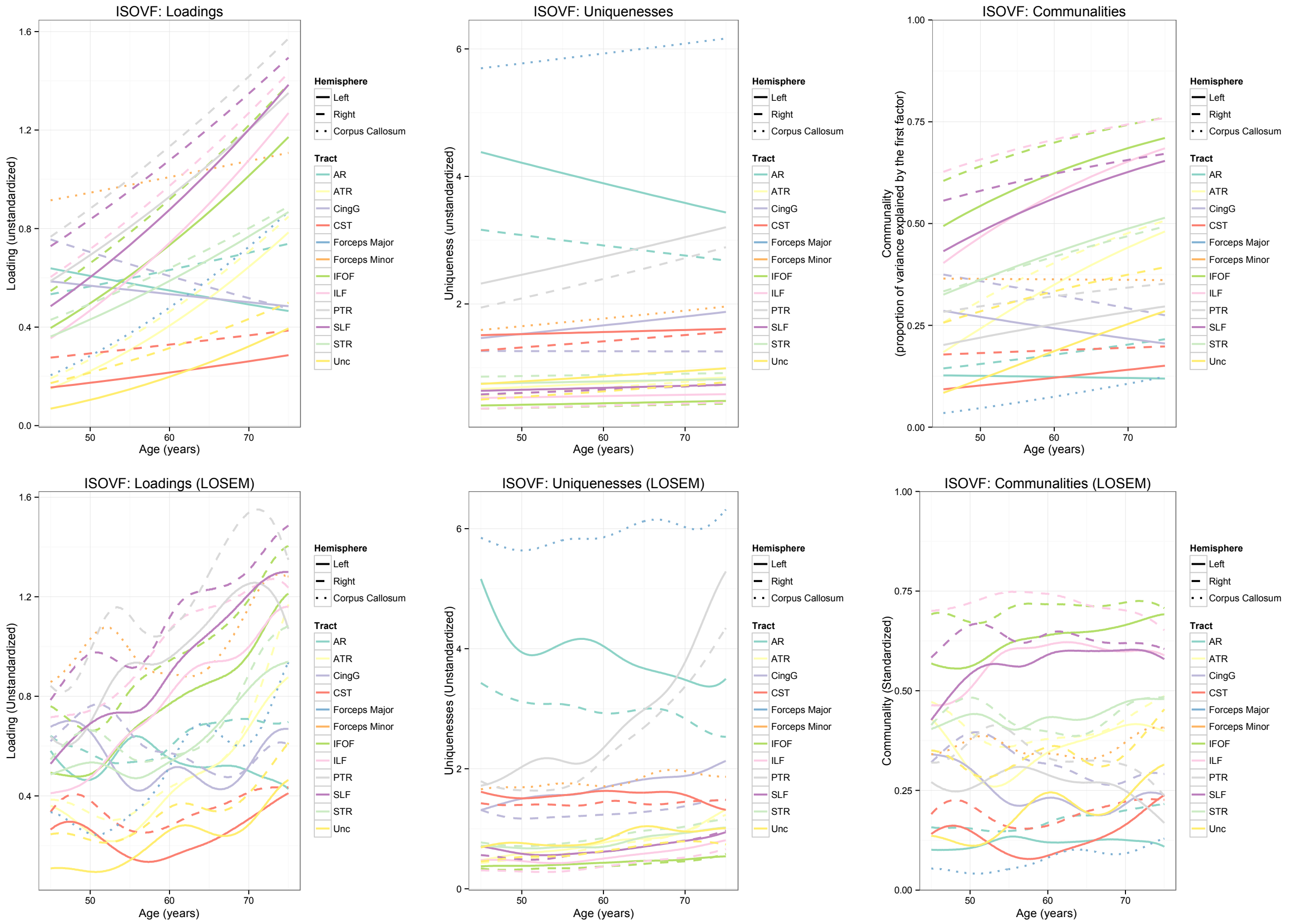

Note. Trajectories for factor loadings (left), tract uniquenesses (centre) and tract communalities (right) for isotropic volume fraction (ISOVF), taken from the multi-parameter age moderation model (top row) and LOSEM model (bottom row).

Supplementary Figure 14. De-differentiation trajectories for OD.

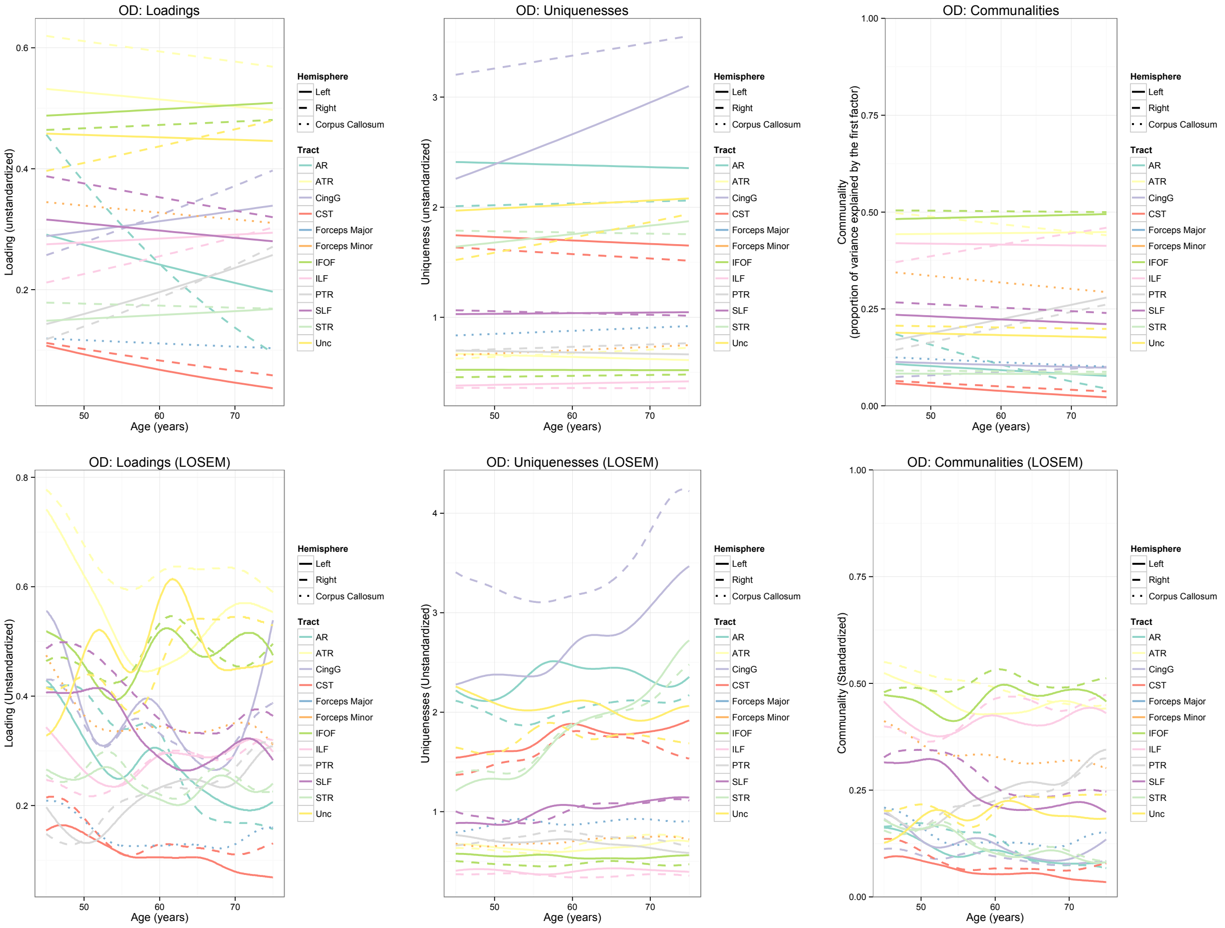

Note. Trajectories for factor loadings (left), tract uniquenesses (centre) and tract communalities (right) for orientation dispersion (OD), taken from the multi-parameter age moderation model (top row) and LOSEM model (bottom row).

Supplementary Figure 15. De-differentiation trajectories for axial diffusivity.

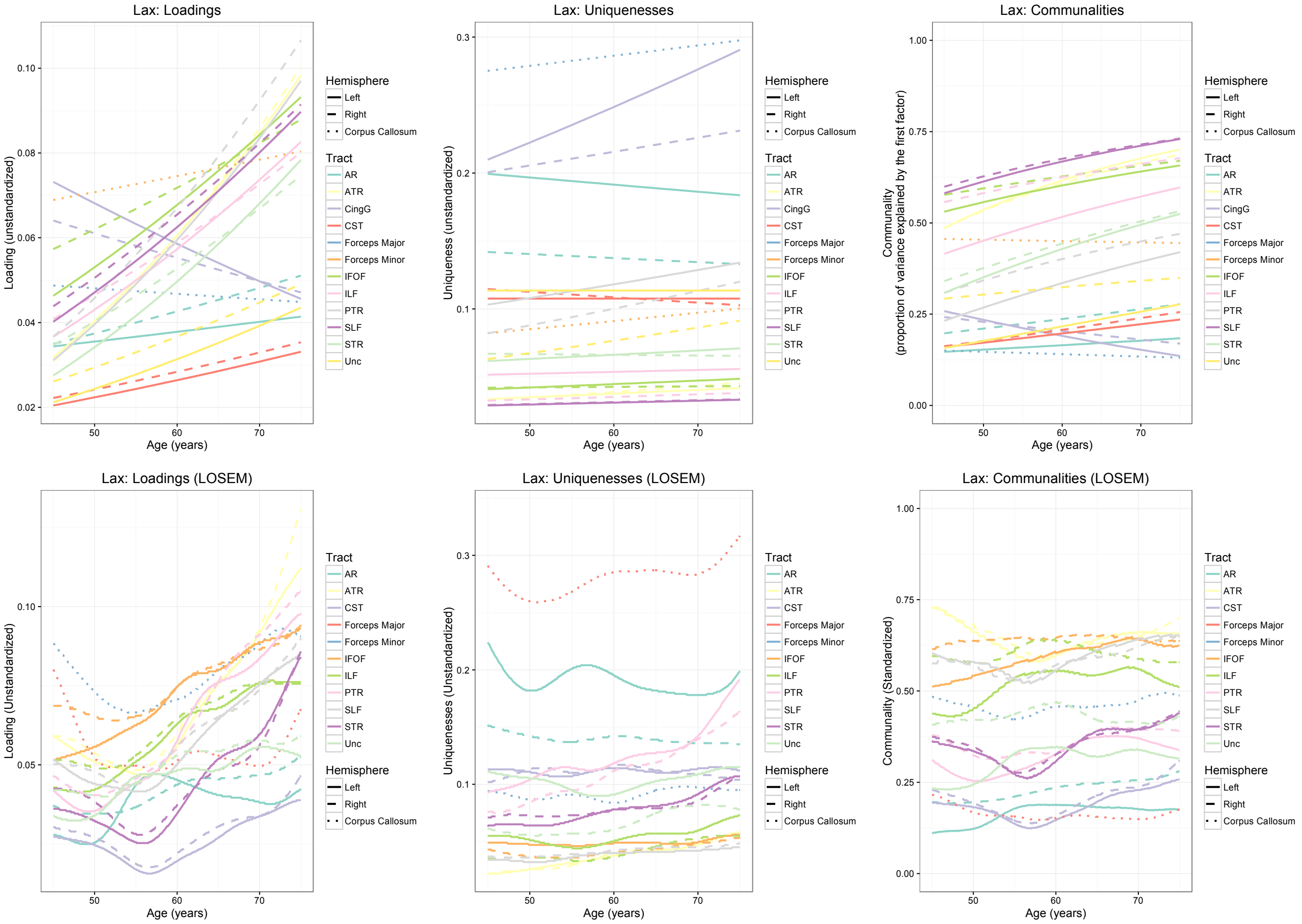

Note. Trajectories for factor loadings (left), tract uniquenesses (centre) and tract communalities (right) for axial diffusivity (λax), taken from the multi-parameter age moderation model (top row) and LOSEM model (bottom row).

Supplementary Figure 16. De-differentiation trajectories for radial diffusivity.

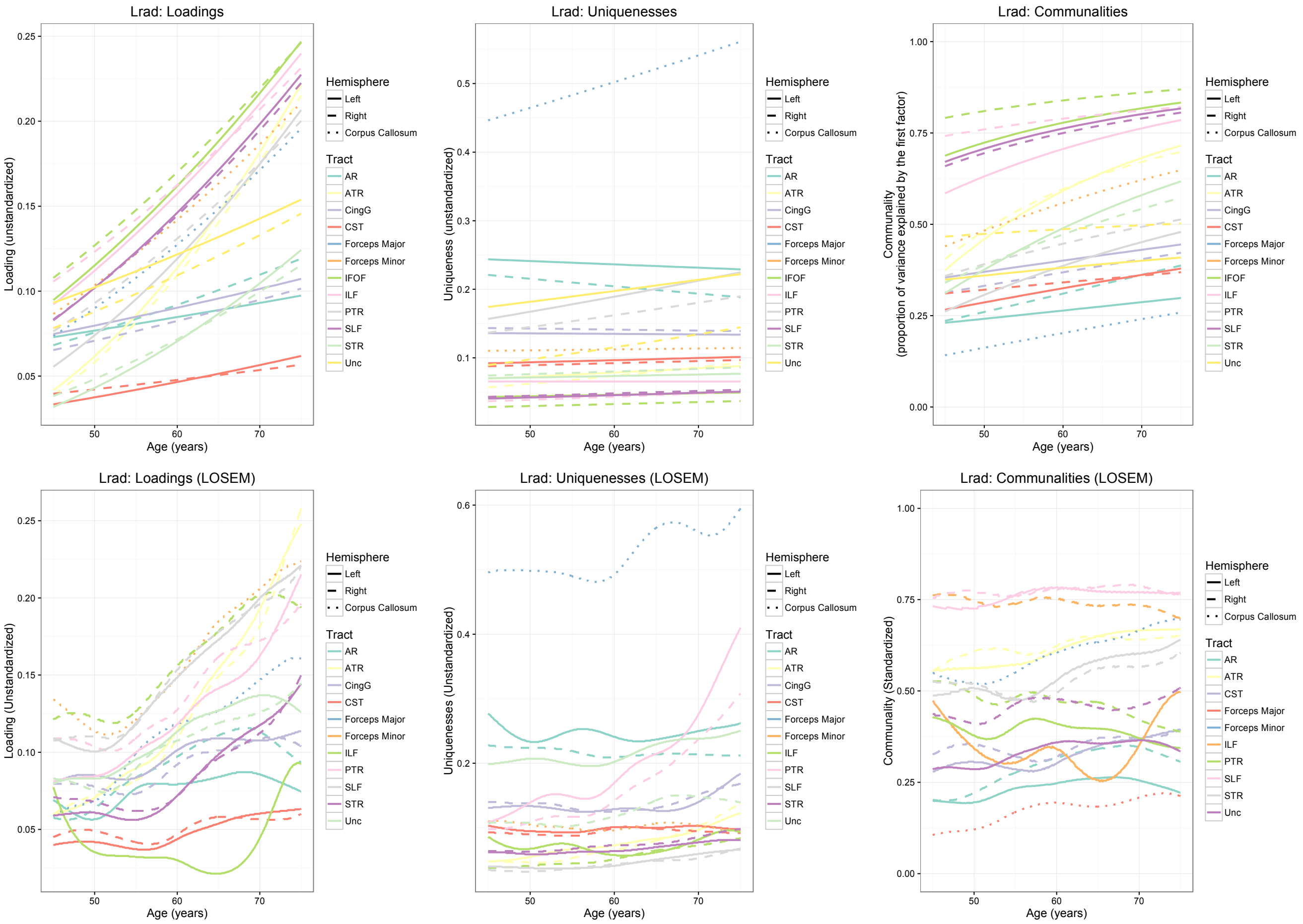

Note. Trajectories for factor loadings (left), tract uniquenesses (centre) and tract communalities (right) for radial diffusivity (λrad), taken from the multi-parameter age moderation model (top row) and LOSEM model (bottom row).

Supplementary Tables

Supplementary Table 1. Characteristics of participants and tract-averaged water diffusion parameters of white matter pathways.

	Variable	Unit	M(SD)	N
Demographics	Age	Years	61.72 (7.47)	3513
	Sex	F:M (%F)	1849:1664 (53%)	3513
	College Degree	Yes (Yes%)	1493 (43%)	3511
	Heart or cardiac problem	Yes (Yes%)	9 (<1%)	3511
	Diagnosis of diabetes	Yes (Yes%)	99 (3%)	3511
	Hypertension	Yes (Yes%)	697 (20%)	3511
	Ethnicity	W:M:O	3419:13:79	3511
	Handedness	R:L:B	3137:314:60	3511
Association Fibres	Inferior Fronto-Occipital Fasciculus	FA	0.472 (0.020)	3512
		MD ($\times 10^{-3}$ mm^2/s)	0.805 (0.030)	3511
	Inferior Longitudinal Fasciculus	FA	0.458 (0.019)	3513
		MD ($\times 10^{-3}$ mm^2/s)	0.815 (0.031)	3513
	Superior Longitudinal Fasciculus	FA	0.435 (0.020)	3513
		MD ($\times 10^{-3}$ mm^2/s)	0.746 (0.029)	3513
	Uncinate Fasciculus	FA	0.393 (0.020)	3510
		MD ($\times 10^{-3}$ mm^2/s)	0.791 (0.033)	3511
	Cingulum (Gyrus)	FA	0.519 (0.029)	3513
		MD ($\times 10^{-3}$ mm^2/s)	0.756 (0.026)	3513
	Cingulum (Parahippocampal)	FA	0.311 (0.029)	3513
		MD ($\times 10^{-3}$ mm^2/s)	0.881 (0.055)	3512
Thalamic Projections	Anterior Thalamic Radiation	FA	0.398 (0.017)	3513
		MD ($\times 10^{-3}$ mm^2/s)	0.774 (0.033)	3512
	Posterior Thalamic Radiation	FA	0.456 (0.019)	3513
		MD ($\times 10^{-3}$ mm^2/s)	0.842 (0.038)	3513
	Superior Thalamic Radiation	FA	0.423 (0.020)	3513
		MD ($\times 10^{-3}$ mm^2/s)	0.754 (0.275)	3513
Sensory Projections	Acoustic Radiation	FA	0.421 (0.019)	3513
		MD ($\times 10^{-3}$ mm^2/s)	0.782 (0.032)	3513
	Corticospinal Tract	FA	0.545 (0.021)	3513
		MD ($\times 10^{-3}$ mm^2/s)	0.772 (0.219)	3513
	Middle Cerebellar Peduncle	FA	0.475 (0.033)	3512
		MD ($\times 10^{-3}$ mm^2/s)	0.774 (0.067)	3512
	Medial Lemniscus	FA	0.419 (0.020)	3513
		MD ($\times 10^{-3}$ mm^2/s)	0.906 (0.034)	3512
Callosal Fibres	Forceps Major	FA	0.585 (0.026)	3513
		MD ($\times 10^{-3}$ mm^2/s)	0.892 (0.050)	3513
	Forceps Minor	FA	0.467 (0.021)	3513
		MD ($\times 10^{-3}$ mm^2/s)	0.829 (0.034)	3510

Note. Tract values are reported for average of left and right. FA: fractional anisotropy, MD: mean diffusivity, ethnicity reported as white:mixed:other, handedness reported as right:left:both.

Supplementary Table 2. Tract-averaged NODDI characteristics of white matter pathways.

	**Variable**	**Unit**	**M(SD)**	**N**
Association Fibres	Inferior Fronto-Occipital Fasciculus	ICVF	0.527 (0.036)	3512
		ISOVF	0.061 (0.011)	3511
		OD	0.168 (0.009)	3512
	Inferior Longitudinal Fasciculus	ICVF	0.519 (0.031)	3513
		ISOVF	0.067 (0.012)	3513
		OD	0.184 (0.007)	3513
	Superior Longitudinal Fasciculus	ICVF	0.617 (0.033)	3513
		ISOVF	0.077 (0.013)	3513
		OD	0.227 (0.011)	3513
	Uncinate Fasciculus	ICVF	0.476 (0.027)	3513
		ISOVF	0.031 (0.009)	3512
		OD	0.231 (0.013)	3511
	Cingulum (Gyrus)	ICVF	0.558 (0.032)	3513
		ISOVF	0.045 (0.013)	3513
		OD	0.137 (0.016)	3513
	Cingulum (Parahippocampal)	ICVF	0.450 (0.023)	3513
		ISOVF	0.080 (0.031)	3513
		OD	0.290 (0.025)	3513
Thalamic Projections	Anterior Thalamic Radiation	ICVF	0.540 (0.029)	3512
		ISOVF	0.055 (0.011)	3513
		OD	0.240 (0.011)	3512
	Posterior Thalamic Radiation	ICVF	0.518 (0.029)	3513
		ISOVF	0.086 (0.019)	3513
		OD	0.185 (0.009)	3513
	Superior Thalamic Radiation	ICVF	0.605 (0.026)	3513
		ISOVF	0.076 (0.012)	3513
		OD	0.248 (0.015)	3513
Sensory Projections	Acoustic Radiation	ICVF	0.548 (0.026)	3513
		ISOVF	0.068 (0.017)	3513
		OD	0.247 (0.013)	3513
	Corticospinal Tract	ICVF	0.682 (0.022)	3513
		ISOVF	0.116 (0.012)	3513
		OD	0.157 (0.013)	3513
	Middle Cerebellar Peduncle	ICVF	0.733 (0.028)	3512
		ISOVF	0.139 (0.037)	3512
		OD	0.233 (0.022)	3512
	Medial Lemniscus	ICVF	0.606 (0.021)	3512
		ISOVF	0.167 (0.022)	3512
		OD	0.184 (0.012)	3512
Callosal Fibres	Forceps Major	ICVF	0.598 (0.030)	3513
		ISOVF	0.141 (0.025)	3513
		OD	0.127 (0.010)	3513
	Forceps Minor	ICVF	0.549 (0.036)	3513
		ISOVF	0.089 (0.017)	3513
		OD	0.196 (0.010)	3513

Note. NODDI: Neurite orientation dispersion and density imaging, ICVF: intracellular volume fraction, ISOVF: isotropic volume fraction, OD: orientation dispersion.

Supplementary Table 3. Tract-averaged axial, radial and MO characteristics of white matter pathways.

	Variable	Unit	M(SD)	N
Association Fibres	Inferior Fronto-Occipital Fasciculus	λax (×10^{-2})	0.126 (0.003)	3512
		λrad (×10^{-3})	0.916 (0.048)	3512
		MO	0.538 (0.031)	3512
	Inferior Longitudinal Fasciculus	λax (×10^{-2})	0.126 (0.003)	3513
		λrad (×10^{-3})	0.941 (0.049)	3513
		MO	0.491 (0.030)	3513
	Superior Longitudinal Fasciculus	λax (×10^{-2})	0.111 (0.003)	3513
		λrad (×10^{-3})	0.904 (0.046)	3513
		MO	0.307 (0.040)	3513
	Uncinate Fasciculus	λax (×10^{-2})	0.115 (0.004)	3510
		λrad (×10^{-3})	0.962 (0.052)	3510
		MO	0.493 (0.035)	3506
	Cingulum (Gyrus)	λax (×10^{-2})	0.126 (0.005)	3513
		λrad (×10^{-3})	0.806 (0.046)	3513
		MO	0.608 (0.057)	3513
	Cingulum (Parahippocampal)	λax (×10^{-2})	0.118 (0.005)	3513
		λrad (×10^{-3})	1.140 (0.089)	3513
		MO	0.427 (0.054)	3513
Thalamic Projections	Anterior Thalamic Radiation	λax (×10^{-2})	0.112 (0.004)	3513
		λrad (×10^{-3})	0.948 (0.050)	3513
		MO	0.410 (0.037)	3513
	Posterior Thalamic Radiation	λax (×10^{-2})	0.130 (0.004)	3513
		λrad (×10^{-3})	0.971 (0.058)	3513
		MO	0.484 (0.040)	3512
	Superior Thalamic Radiation	λax (×10^{-2})	0.112 (0.004)	3513
		λrad (×10^{-3})	0.911 (0.040)	3513
		MO	0.404 (0.054)	3511
Sensory Projections	Acoustic Radiation	λax (×10^{-2})	0.115 (0.004)	3513
		λrad (×10^{-3})	0.953 (0.049)	3513
		MO	0.354 (0.048)	3513
	Corticospinal Tract	λax (×10^{-2})	0.131 (0.004)	3513
		λrad (×10^{-3})	0.810 (0.036)	3513
		MO	0.634 (0.041)	3513
	Middle Cerebellar Peduncle	λax (×10^{-2})	0.120 (0.009)	3512
		λrad (×10^{-3})	0.888 (0.091)	3512
		MO	0.499 (0.068)	3510
	Medial Lemniscus	λax (×10^{-2})	0.140 (0.004)	3513
		λrad (×10^{-3})	1.035 (0.053)	3513
		MO	0.670 (0.038)	3510
Callosal Fibres	Forceps Major	λax (×10^{-2})	0.157 (0.006)	3513
		λrad (×10^{-3})	0.873 (0.081)	3513
		MO	0.729 (0.033)	3510
	Forceps Minor	λax (×10^{-2})	0.130 (0.004)	3513
		λrad (×10^{-3})	0.943 (0.053)	3513
		MO	0.494 (0.038)	3513

Note. λax = axial diffusivity, λrad = radial diffusivity, MO = diffusion tensor mode.

Supplementary Table 4. Regression models of age and sex on tract-averaged white matter FA and MD.

	Tract		Age	Age^2	Sex	Hemisphere	Sex*Age	R^2
Association Fibres	IFOF	FA†	**-0.221 (< 0.001)**	-0.023 (0.035)	0.007 (0.523)	**-0.264 (< 0.001)**	0.017 (0.128)	0.116
		MD†	**0.338 (< 0.001)**	**0.070 (< 0.001)**	0.034 (0.002)	**0.129 (< 0.001)**	-0.009 (0.404)	0.127
	ILF	FA†	**-0.252 (< 0.001)**	-0.025 (0.022)	-0.012 (0.293)	**-0.249 (< 0.001)**	0.016 (0.162)	0.123
		MD†	**0.306 (< 0.001)**	**0.058 (< 0.001)**	**0.053 (< 0.001)**	**0.138 (< 0.001)**	-0.011 (0.326)	0.112
	SLF	FA†	**-0.213 (< 0.001)**	-0.030 (0.005)	0.031 (0.005)	**-0.386 (< 0.001)**	**0.042 (< 0.001)**	0.193
		MD†	**0.312 (< 0.001)**	**0.061 (< 0.001)**	-0.009 (0.415)	**0.175 (< 0.001)**	-0.037 (0.001)	0.123
	Uncinate	FA	**-0.219 (< 0.001)**		**0.134 (0.001)**	-0.025 (0.028)	0.006 (0.623)	0.061
		MD†	**0.321 (< 0.001)**	**0.058 (< 0.001)**	-0.011 (0.334)	**0.055 (< 0.001)**	-0.001 (0.964)	0.099
	Cingulum (Gyrus)	FA	**-0.132 (< 0.001)**		**0.137 (< 0.001)**	**-0.511 (< 0.001)**	0.001 (0.898)	0.294
		MD	**0.139 (< 0.001)**		**-0.081 (< 0.001)**	**0.068 (< 0.001)**	**-0.053 (< 0.001)**	0.031
	Cingulum (PH)	FA	-0.036 (0.002)		**0.202 (< 0.001)**	-0.02 (0.042)	**-0.042 (< 0.001)**	0.043
		MD†	**0.165 (< 0.001)**	**0.062 (< 0.001)**	**0.059 (< 0.001)**	**0.083 (< 0.001)**	0.025 (0.031)	0.038
Thalamic Projections	ATR	FA	**-0.255 (< 0.001)**		**0.106 (<0.001)**	**-0.215 (< 0.001)**	0.035 (0.002)	0.119
		MD†	**0.496 (< 0.001)**	**0.103 (< 0.001)**	**0.056 (< 0.001)**	0.003 (0.783)	-0.003 (0.752)	0.238
	PTR	FA†	**-0.226 (< 0.001)**	**-0.039 (< 0.001)**	0.002 (0.835)	**-0.113 (< 0.001)**	-0.018 (0.132)	0.061
		MD†	**0.362 (< 0.001)**	**0.096 (< 0.001)**	**0.104 (< 0.001)**	**0.044 (< 0.001)**	**0.038 (<0.001)**	0.144
	STR	FA	-0.028 (0.016)		**0.190 (< 0.001)**	**-0.040 (< 0.001)**	**0.067 (< 0.001)**	0.042
		MD†	**0.361 (< 0.001)**	**0.057 (< 0.001)**	-0.034 (0.002)	**-0.060 (< 0.001)**	0.007 (0.519)	0.126
Sensory Projections	AR	FA	-0.036 (0.002)		**0.170 (< 0.001)**	**-0.232 (< 0.001)**	0.020 (0.082)	0.083
		MD	**0.043 (< 0.001)**		**-0.114 (0.001)**	**0.047 (< 0.001)**	0.005 (0.701)	0.016
	Corticospinal Tract	FA	**-0.086 (< 0.001)**		**0.218 (< 0.001)**	**-0.151 (< 0.001)**	**0.079 (< 0.001)**	0.080
		MD†	**0.155 (< 0.001)**	**0.039 (< 0.001)**	-0.033 (0.005)	**0.093 (< 0.001)**	**0.054 (< 0.001)**	0.034
	MCP	FA	-0.047 (0.005)		**0.171 (0.001)**	-	0.006 (0.734)	0.030
		MD	**0.152 (< 0.001)**		**0.103 (< 0.001)**	-	**0.072 (< 0.001)**	0.041
	Medial Lemniscus	FA	-0.019 (0.113)		**0.193 (< 0.001)**	**0.065 (< 0.001)**	0.035 (0.003)	0.042
		MD	**0.045 (< 0.001)**		**-0.110 (< 0.001)**	0.038 (0.001)	0.039 (0.001)	0.016
Callosal Fibres	Forceps Major	FA†	**-0.061 (< 0.001)**	-0.041 (0.013)	0.027 (0.109)	-	0.012 (0.488)	0.004
		MD	**0.059 (< 0.001)**		0.025 (0.132)	-	-0.017 (0.323)	0.004
	Forceps Minor	FA	**-0.275 (< 0.001)**		**0.083 (< 0.001)**	-	0.019 (0.239)	0.078
		MD†	**0.199 (< 0.001)**	0.041 (0.012)	-0.001 (0.932)	-	-0.001 (0.975)	0.036

Note. Standardised βs (p values) and adjusted R^2 reported. †quadratic model significantly better fit ($p < 0.05$). Bold type indicates significant effect ($p < 0.001$). AR: acoustic radiation, ATR: anterior thalamic radiation, IFOF: inferior fronto-occipital fasciculus, ILF: inferior longitudinal fasciculus, MCP: middle cerebellar peduncle, PH: parahippocampal portion, PTR: posterior thalamic radiation, SLF: superior longitudinal fasciculus, STR: superior thalamic radiation, FA: fractional anisotropy, MD: mean diffusivity. Female and left hemisphere coded as 0.

Supplementary Table 5. Regression models of age and sex on tract-averaged white matter ICVF, ISOVF and OD.

	**Tract**		**Age**	**Age^2**	**Sex**	**Hemisphere**	**Sex*Age**	***R^2***
Association Fibres	IFOF	ICVF†	**-0.251 (< 0.001)**	**-0.047 (< 0.001)**	**-0.039 (< 0.001)**	0.032 (0.005)	0.020 (0.081)	0.063
		ISOVF†	**0.205 (< 0.001)**	**0.046 (< 0.001)**	-0.014 (0.206)	**0.326 (< 0.001)**	0.023 (0.043)	0.145
		OD†	**-0.062 (< 0.001)**	-0.036 (0.002)	**-0.080 (< 0.001)**	**0.247 (< 0.001)**	0.010 (0.372)	0.071
	ILF	ICVF†	**-0.021 (< 0.001)**	**-0.044 (<0.001)**	**-0.060 (< 0.001)**	-0.007 (0.564)	0.022 (0.067)	0.045
		ISOVF†	**0.241 (< 0.001)**	0.0346 (0.002)	-0.020 (0.072)	**0.287 (< 0.001)**	0.017 (0.122)	0.137
		OD†	0.009 (0.453)	-0.034 (0.003)	**-0.111 (< 0.001)**	**0.225 (<0.001)**	0.017 (0.154)	0.064
	SLF	ICVF†	**-0.227 (< 0.001)**	**-0.058 (< 0.001)**	-0.016 (0.181)	**-0.090 (< 0.001)**	0.036 (0.002)	0.057
		ISOVF	**0.256 (< 0.001)**		-0.021 (0.061)	**0.237 (< 0.001)**	-0.014 (0.198)	0.121
		OD†	**-0.050 (< 0.001)**	-0.034 (0.002)	**-.102 (< 0.001)**	**0.361 (< 0.001)**	0.014 (0.201)	0.144
	Uncinate	ICVF†	**-0.282 (< 0.001)**	**-0.067 (< 0.001)**	**0.074 (< 0.001)**	0.019 (0.091)	0.003 (0.778)	0.077
		ISOVF	**0.200 (< 0.001)**		**0.062 (< 0.001)**	0.020 (0.084)	0.018 (0.117)	0.046
		OD†	0.022 (0.072)	-0.025 (0.030)	**-0.137 (< 0.001)**	**-0.169 (< 0.001)**	0.002 (0.861)	0.047
	Cingulum (Gyrus)	ICVF†	**-0.212 (< 0.001)**	**-0.050 (< 0.001)**	**0.140 (< 0.001)**	**-0.344 (< 0.001)**	0.010 (0.378)	0.175
		ISOVF†	**-0.058 (< 0.001)**	-0.035 (0.001)	**0.089 (< 0.001)**	**-0.319 (< 0.001)**	**-0.041 (< 0.001)**	0.114
		OD	**0.083 (< 0.001)**		**-0.069 (< 0.001)**	**0.358 (< 0.001)**	0.023 (0.037)	0.139
	Cingulum (PH)	ICVF†	**-0.047 (< 0.001)**	**-0.051 (< 0.001)**	**0.191 (< 0.001)**	0.034 (<0.003)	-0.011 (0.365)	0.040
		ISOVF†	**0.160 (< 0.001)**	**0.045 (< 0.001)**	**0.117 (< 0.001)**	**0.089 (< 0.001)**	0.029 (0.014)	0.049
		OD	0.024 (0.040)		**-0.157 (< 0.001)**	-0.029 (0.015)	**0.039 (< 0.001)**	0.026
Thalamic Projections	ATR	ICVF†	**-0.382 (< 0.001)**	**-0.070 (< 0.001)**	0.027 (0.015)	-0.017 (0.124)	0.001 (0.908)	0.136
		ISOVF†	**0.343 (< 0.001)**	**0.089 (< 0.001)**	**0.135 (< 0.001)**	**-0.061 (< 0.001)**	0.009 (0.428)	0.139
		OD†	**-0.197 (< 0.001)**	**-0.077 (< 0.001)**	**-0.125 (< 0.001)**	**0.215 (< 0.001)**	-0.011 (0.346)	0.103
	PTR	ICVF†	**-0.220 (< 0.001)**	-0.033 (0.004)	**-0.108 (< 0.001)**	**0.083 (< 0.001)**	-0.004 (0.714)	0.068
		ISOVF†	**0.285 (< 0.001)**	**0.093 (< 0.001)**	0.019 (0.091)	**0.175 (< 0.001)**	**0.042 (< 0.001)**	0.111
		OD†	**-0.103 (< 0.001)**	-0.029 (0.010)	**-0.163 (< 0.001)**	**0.119 (< 0.001)**	0.007 (0.524)	0.053
	STR	ICVF†	**-0.260 (< 0.001)**	**-0.048 (< 0.001)**	-0.012 (0.282)	**0.056 (< 0.001)**	0.012 (0.301)	0.067
		ISOVF†	**0.295 (< 0.001)**	0.032 (0.005)	**-0.065 (< 0.001)**	-0.027 (0.017)	0.008 (0.467)	0.085
		OD†	**-0.277 (< 0.001)**	**-0.069 (< 0.001)**	**-0.255 (< 0.001)**	**-0.038 (< 0.001)**	-0.044 (< 0.001)	0.151
Motor & Sensory Projections	AR	ICVF†	**-0.161 (< 0.001)**	**-0.048 (< 0.001)**	**0.084 (< 0.001)**	-0.019 (0.103)	0.018 (0.126)	0.029
		ISOVF†	-0.031 (0.011)	-0.029 (0.012)	**-0.064 (< 0.001)**	**0.072 (< 0.001)**	0.021 (0.084)	0.011
		OD†	**-0.107 (< 0.001)**	**-0.047 (< 0.001)**	**-0.096 (< 0.001)**	**0.270 (< 0.001)**	0.006 (0.628)	0.095
	Corticospinal Tract	ICVF†	**-0.102 (< 0.001)**	**-0.045 (< 0.001)**	**0.107 (< 0.001)**	**-0.161 (< 0.001)**	0.009 (0.454)	0.045
		ISOVF	**0.153 (< 0.001)**		0.006 (0.583)	**-0.019 (0.111)**	**0.048 (< 0.001)**	0.026
		OD†	**-0.060 (< 0.001)**	-0.036 (0.001)	**-0.261 (< 0.001)**	0.013 (0.241)	**-0.050 (< 0.001)**	0.077
	MCP	ICVF†	**-0.057 (< 0.001)**	-0.057 (0.004)	**0.196 (< 0.001)**	-	-0.002 (0.925)	0.040
		ISOVF	**0.146 (< 0.001)**		0.016 (0.330)	-	**0.069 (< 0.001)**	0.049
		OD	-0.003 (0.047)		**-0.184 (< 0.001)**	-	-0.012 (0.483)	0.035
	Medial Lemniscus	ICVF	0.016 (0.165)		**0.242 (< 0.001)**	**-0.053 (< 0.001)**	-0.001 (0.916)	0.061
		ISOVF	0.037 (0.002)		-0.030 (0.013)	-0.003 (0.828)	0.037 (0.002)	0.003
		OD	0.023 (0.048)		**-0.156 (< 0.001)**	-0.008 (0.503)	-0.031 (0.009)	0.025
Callosal Fibres	Forceps Major	ICVF†	**-0.100 (< 0.001)**	-0.042 (0.011)	**-0.076 (< 0.001)**	-	0.021 (0.223)	0.016
		ISOVF	0.029 (0.089)		-0.024 (0.164)	-	-0.016 (0.333)	0.001
		OD	-0.049 (0.003)		**-0.200 (< 0.001)**	-	0.020 (0.221)	0.044
	Forceps Minor	ICVF†	**-0.281 (< 0.001)**	-0.032 (0.044)	-0.004 (0.817)	-	0.024 (0.136)	0.075
		ISOVF	-0.050 (0.003)		-0.009 (0.603)	-	0.030 (0.079)	0.003
		OD	0.042 (0.013)		**-0.140 (< 0.001)**	-	0.008 (0.621)	0.020

Note. Standardised βs (p values) and adjusted R^2 reported. †quadratic model significantly better fit ($p < 0.05$). Bold type indicates significant ($p < 0.001$). AR: acoustic radiation, ATR: anterior thalamic radiation, IFOF: inferior fronto-occipital fasciculus, ILF: inferior longitudinal fasciculus, MCP: middle cerebellar peduncle, PH: parahippocampal portion, PTR: posterior thalamic radiation, SLF: superior longitudinal fasciculus, STR: superior thalamic radiation, FA: fractional anisotropy, MD: mean diffusivity. Female and left hemisphere coded as 0.

Supplementary Table 6. Regression models of age and sex on tract-averaged white matter λax, λrad and MO.

	Tract		Age	Age^2	Sex	Hemisphere	Sex*Age	R^2
Association Fibres	IFOF	λax†	**0.268 (< 0.001)**	**0.070 (< 0.001)**	**0.059 (< 0.001)**	-0.019 (0.096)	-0.010 (0.407)	0.073
		λrad†	**0.331 (< 0.001)**	**0.062 (< 0.001)**	0.011 (0.340)	**0.183 (< 0.001)**	-0.008 (0.448)	0.136
		MO	**-0.078 (< 0.001)**		**0.072 (< 0.001)**	**-0.075 (< 0.001)**	-0.028 (0.020)	0.016
	ILF	λax†	**0.235 (< 0.001)**	**0.058 (< 0.001)**	**0.077 (< 0.001)**	0.008 (0.505)	-0.012 (0.291)	0.060
		λrad†	**0.314 (< 0.001)**	**0.054 (< 0.001)**	0.026 (0.022)	**0.195 (< 0.001)**	-0.008 (0.463)	0.132
		MO†	**-0.105 (< 0.001)**	-0.027 (0.015)	**0.170 (< 0.001)**	**-0.251 (< 0.001)**	-0.020 (0.082)	0.099
	SLF	λax†	**0.277 (< 0.001)**	**0.063 (< 0.001)**	0.029 (0.011)	-0.013 (0.254)	-0.023 (0.045)	0.074
		λrad†	**0.299 (< 0.001)**	**0.055 (< 0.001)**	**-0.041 (< 0.001)**	**0.224 (< 0.001)**	**-0.041 (< 0.001)**	0.135
		MO	-0.001 (0.916)		**0.215 (< 0.001)**	**0.116 (< 0.001)**	0.002 (0.167)	0.060
	Uncinate	λax†	**0.266 (< 0.001)**	**0.059 (< 0.001)**	**0.066 (< 0.001)**	**0.113 (< 0.001)**	0.003 (0.819)	0.086
		λrad†	**0.320 (< 0.001)**	**0.050 (< 0.001)**	**-0.048 (< 0.001)**	0.009 (0.441)	-0.002 (0.882)	0.096
		MO	**-0.047 (< 0.001)**		**0.056 (< 0.001)**	**0.274 (< 0.001)**	0.002 (0.836)	0.080
	Cingulum (Gyrus)	λax	-0.027 (0.011)		**0.049 (< 0.001)**	**-0.429 (<0.001)**	**-0.042 (< 0.001)**	0.189
		λrad†	**0.202 (< 0.001)**	0.031 (0.002)	**-0.127 (< 0.001)**	**0.450 (< 0.001)**	-0.026 (0.012)	0.253
		MO†	**-0.235 (< 0.001)**	**-0.056 (< 0.001)**	**0.054 (< 0.001)**	**-0.496 (< 0.001)**	-0.020 (0.052)	0.299
	Cingulum (PH)	λax†	**0.168 (<0.001)**	**0.065 (< 0.001)**	**0.169 (< 0.001)**	**0.098 (< 0.001)**	0.008 (0.496)	0.070
		λrad†	**0.153 (< 0.001)**	**0.054 (< 0.001)**	0.011 (0.371)	**0.067 (< 0.001)**	0.032 (0.007)	0.028
		MO	-0.018 (0.115)		**0.157 (< 0.001)**	**0.131 (< 0.001)**	-0.009 (0.434)	0.041
Thalamic Projections	ATR	λax†	**0.478 (< 0.001)**	**0.115 (< 0.001)**	**0.126 (< 0.001)**	**-0.076 (< 0.001)**	0.007 (0.532)	0.246
		λrad†	**0.468 (< 0.001)**	**0.090 (< 0.001)**	**0.016 (0.125)**	0.030 (0.005)	-0.009 (0.419)	0.208
		MO	-0.001 (0.924)		**0.085 (< 0.001)**	0.029 (0.014)	-0.029 (0.016)	0.008
	PTR	λax†	**0.300 (< 0.001)**	**0.093 (< 0.001)**	**0.139 (< 0.001)**	-0.032 (0.004)	0.028 (0.014)	0.113
		λrad†	**0.371 (< 0.001)**	**0.092 (< 0.001)**	**0.066 (<0.001)**	**0.087 (<0.001)**	**0.043 (< 0.001)**	0.147
		MO†	**-0.129 (< 0.001)**	-0.028 (0.016)	**0.150 (< 0.001)**	**-0.060 (< 0.001)**	**-0.055 (< 0.001)**	0.041
	STR	λax†	**0.388 (< 0.001)**	**0.075 (< 0.001)**	**0.087 (0.001)**	**-0.061 (<0.001)**	**0.038 (<0.001)**	0.160
		λrad†	**0.274 (< 0.001)**	0.035 (0.002)	**-0.121 (< 0.001)**	**-0.068 (< 0.001)**	-0.017 (0.144)	0.085
		MO†	**0.212 (< 0.001)**	0.032 (< 0.003)	**0.240 (< 0.001)**	**0.166 (< 0.001)**	**0.038 (< 0.001)**	0.137
Motor & Sensory Projections	AR	λax	0.036 (0.002)		**-0.047 (< 0.001)**	**-0.103 (< 0.001)**	0.011 (0.335)	0.013
		λrad	**0.043 (< 0.001)**		**-0.135 (< 0.001)**	**0.147 (< 0.001)**	-0.002 (0.873)	0.040
		MO	-0.015 (0.202)		-0.006 (0.588)	**-0.301 (< 0.001)**	0.024 (0.036)	0.091
	Corticospinal Tract	λax†	**0.099 (< 0.001)**	**0.048 (< 0.001)**	**0.048 (< 0.001)**	**0.138 (< 0.001)**	-0.024 (0.037)	0.041
		λrad	**0.130 (<0.001)**		-0.148 (< 0.001)	**0.013 (< 0.001)**	0.003 (0.779)	0.051
		MO	0.014 (0.243)		**0.140 (< 0.001)**	0.046 (0.001)	**0.067 (< 0.001)**	0.026
	MCP	λax	**0.134 (< 0.001)**		**0.152 (< 0.001)**	-	**0.064 (< 0.001)**	0.048
		λrad	**0.150 (< 0.001)**		0.054 (0.001)	-	**0.071 (< 0.001)**	0.031
		MO	0.012 (0.483)		**0.174 (< 0.001)**	-	0.045 (0.007)	0.032
	Medial Lemniscus	λax	**0.060 (< 0.001)**		0.034 (0.005)	0.020 (0.089)	**0.047 (< 0.001)**	0.007
		λrad	0.035 (0.003)		-0.185 (< 0.001)	0.038 (0.001)	0.024 (0.044)	0.036
		MO†	-0.027 (0.026)	-0.030 (0.009)	**0.153 (< 0.001)**	**0.047 (< 0.001)**	0.022 (0.069)	0.026
Callosal Fibres	Forceps Major	λax	**0.064 (< 0.001)**		**0.056 (< 0.001)**	-	-0.021 (0.218)	0.007
		λrad†	0.057 (0.001)	0.035 (0.035)	0.002 (0.926)	-	-0.015 (0.384)	0.003
		MO†	**-0.100 (< 0.001)**	**-0.059 (< 0.001)**	**0.073 (< 0.001)**	-	-0.032 (0.059)	0.015
	Forceps Minor	λax†	**0.094 (< 0.001)**	0.046 (0.004)	0.074 (< 0.001)	-	0.009 (0.585)	0.015
		λrad†	0.226 (< 0.001)	0.036 (0.027)	-0.044 (0.008)	-	-0.006 (0.735)	0.048
		MO	**-0.203 (< 0.001)**		-0.020 (0.228)	-	-0.005 (0.772)	0.041

Note. Standardised *β*s (*p* values) and adjusted R^2 reported. †quadratic model significantly better fit ($p < 0.05$). Bold type indicates significant ($p < 0.001$). λax = axial diffusivity, λrad = radial diffusivity, MO = diffusion tensor mode, AR: acoustic radiation, ATR: anterior thalamic radiation, IFOF: inferior fronto-occipital fasciculus, ILF: inferior longitudinal fasciculus, MCP: middle cerebellar peduncle, PH: parahippocampal portion, PTR: posterior thalamic radiation, SLF: superior longitudinal fasciculus, STR: superior thalamic radiation, FA: fractional anisotropy, MD: mean diffusivity. Female and left hemisphere coded as 0.

Supplementary Table 7. Fit statistics for one-factor models for each of the five white matter measurements. All models included 22 tracts. All models were adjusted for age, age^2 (for all except FA), and sex, include residual covariances between the left and right tracts measured bilaterally and additional residual covariances as indicated by the model modification indices.

**Measure**	χ^2	**df**	p	**RMSEA**	**CFI**	**TLI**	**SRMR**
FA	2719.02	174	<.001	0.065	0.964	0.943	0.031
MD	6504.57	176	<.001	0.101	0.930	0.882	0.066
ICVF	6781.80	178	<.001	0.103	0.952	0.920	0.031
ISOVF	4714.85	178	<.001	0.085	0.923	0.871	0.052
OD	3084.41	183	<.001	0.067	0.924	0.876	0.064
λax	6083.863	176	<.001	0.098	0.917	0.859	0.068
λrad	6001.114	182	<.001	0.095	0.931	0.887	0.053

Note. FA: fractional anisotropy; MD: mean diffusivity; ICVF: intracellular volume fraction; ISOVF: isotropic volume fraction; OD: orientation dispersion, λax = axial diffusivity, λrad = radial diffusivity. RMSA: Root Mean Square Error of Approximation; CFI: Comparative Fit Index; TLI: Tucker-Lewis Index; SRMR: Standardized Root Mean Square Residual.

Supplementary Table 8. Standardized factor loadings of each white matter tract in the one-factor models.

Tract	Standardized factor loading						
	**FA**	**MD**	**ICVF**	**ISOVF**	**OD**	λax	λrad
Left AR	0.601	0.484	0.848	0.347	0.299	0.420	0.512
Right AR	0.600	0.550	0.841	0.423	0.305	0.499	0.550
Left ATR	0.748	0.698	0.825	0.561	0.655	0.695	0.694
Right ATR	0.736	0.690	0.820	0.598	0.652	0.686	0.698
Left CingG	0.450	0.697	0.720	0.481	0.319	0.414	0.602
Right CingG	0.416	0.694	0.724	0.558	0.296	0.429	0.590
Left CST	0.482	0.586	0.774	0.350	0.184	0.414	0.558
Right CST	0.472	0.606	0.754	0.431	0.210	0.429	0.564
Forceps Major	0.555	0.431	0.792	0.287	0.325	0.392	0.476
Forceps Minor	0.790	0.739	0.881	0.599	0.554	0.673	0.736
Left IFOF	0.832	0.832	0.927	0.784	0.697	0.754	0.841
Right IFOF	0.831	0.852	0.934	0.820	0.699	0.763	0.875
Left ILF	0.782	0.785	0.920	0.748	0.643	0.709	0.812
Right ILF	0.829	0.833	0.931	0.812	0.641	0.743	0.848
Left PTR	0.591	0.589	0.837	0.482	0.474	0.533	0.612
Right PTR	0.599	0.638	0.849	0.541	0.445	0.554	0.628
Left SLF	0.805	0.853	0.906	0.738	0.469	0.745	0.837
Right SLF	0.816	0.845	0.903	0.759	0.498	0.737	0.826
Left STR	0.597	0.708	0.817	0.631	0.266	0.534	0.674
Right STR	0.568	0.702	0.807	0.628	0.275	0.541	0.657
Left Unc	0.625	0.541	0.748	0.439	0.423	0.536	0.592
Right Unc	0.631	0.652	0.793	0.574	0.447	0.623	0.664

Note. Factor loadings are calculated after controlling for age, age^2 (for all except FA) and sex; note that for many tracts, these loadings changed with age – see the de-differentiation analyses. The models included residual covariances between the left and right tracts measured bilaterally, and additional residual covariances as indicated by the model modification indices. AR: acoustic radiation, ATR: anterior thalamic radiation, IFOF: inferior fronto-occipital fasciculus, ILF: inferior longitudinal fasciculus, MCP: middle cerebellar peduncle, PH: parahippocampal portion, PTR: posterior thalamic radiation, SLF: superior longitudinal fasciculus, STR: superior thalamic radiation, FA: fractional anisotropy, MD: mean diffusivity, ICVF: intra-cellular volume fraction, ISOVF: isotropic volume fraction, OD: orientation dispersion, λax = axial diffusivity, λrad = radial diffusivity.

Supplementary Table 9. Residual covariance paths added to the one-factor models for each white matter structural measure.

FA	MD	ICVF	ISOVF	OD	λax	λrad
L ILF ↔ L IFOF	R ILF ↔ R IFOF	L ILF ↔ L IFOF	L ILF ↔ L IFOF	L STR ↔ L CST	L ILF ↔ L IFOF	L ILF ↔ L IFOF
R PTR ↔ R ILF	R PTR ↔ L ILF	L PTR ↔ L ILF	R ILF ↔ R IFOF	R PTR ↔ R ILF	R STR ↔ R SLF	R PTR ↔ L ILF
R STR ↔ R CST	R PTR ↔ R ILF	R ILF ↔ R IFOF	R PTR ↔ R ILF	L ILF ↔ L IFOF	R PTR ↔ R ILF	R PTR ↔ R ILF
L PTR ↔ L ILF	R STR ↔ R SLF	L PTR ↔ L IFOF	L PTR ↔ L IFOF	R STR ↔ R CST	R STR ↔ R CST	R SLF ↔ L ILF
L PTR ↔ L IFOF	R SLF ↔ L ILF	R PTR ↔ R ILF	L PTR ↔ L ILF	L STR ↔ R CST	L PTR ↔ L ILF	L UNC ↔ L ILF
R ILF ↔ R IFOF	L PTR ↔ L ILF	R STR ↔ RCST	R STR ↔ R SLF	R STR ↔ L CST	L PTR ↔ L IFOF	R STR ↔ R SLF
R PTR ↔ R IFOF	F MIN ↔ F MAJ	R STR ↔ R SLF	F MIN ↔ F MAJ	R ILF ↔ R IFOF	R ILF ↔ R IFOF	F MIN ↔ F MAJ
L PTR ↔ R ILF	R ILF ↔ L IFOF	L STR ↔ L CST	L STR ↔ R SLF	R STR ↔ R SLF	L UNC ↔ F MIN	L UNC ↔ L IFOF
L PTR ↔ R IFOF	L PTR ↔ L IFOF	R PTR ↔ F MIN	L STR ↔ L SLF	R PTR ↔ R IFOF	R UNC ↔ F MIN	R PTR ↔ L IFOF
R IFOF ↔ R ATR	R PTR ↔ L IFOF	R PTR ↔ R IFOF	R STR ↔ L SLF	R STR ↔ R ATR	R PTR ↔ R IFOF	R ILF ↔ R IFOF
L SLF ↔ L ILF	L UNC ↔ L IFOF	L STR ↔ R CST	L IFOF ↔ L ATR	R UNC ↔ R ILF	R STR ↔ R ATR	L IFOF ↔ L ATR
L ILF ↔ R IFOF	L UNC ↔ R ILF	L PTR ↔ F MIN	R IFOF ↔ R ATR	L STR ↔ R ATR	R STR ↔ L SLF	L UNC ↔ R ILF
R PTR ↔ L ILF	R IFOF ↔ R ATR	R STR ↔ L CST	R PTR ↔ R IFOF	F MIN ↔ L ATR	R ILF ↔ L IFOF	R STR ↔ R CST
R ILF ↔ L IFOF	L PTR ↔ R ILF	R SLF ↔ R CST	L SLF ↔ L ILF	L SLF ↔ L CST	L ILF ↔ R IFOF	L STR ↔ R SLF
R PTR ↔ L IFOF	L SLF ↔ L IFOF	R ILF ↔ F MIN	L STR ↔ L CST	F MIN ↔ R ATR	L STR ↔ L SLF	R ILF ↔ L IFOF
L ILF ↔ L AR	R STR ↔ R CST	L ILF ↔ F MIN	R STR ↔ R ATR	L IFOF ↔ L ATR	R PTR ↔ L ILF	L UNC ↔ R PTR
L STR ↔ R CST	R UNC ↔ L IFOF	L PTR ↔ R ILF	L UNC ↔ R STR	-	L UNC ↔ L ATR	L UNC ↔ L PTR
R SLF ↔ L IFOF	R UNC ↔ R ILF	R PTR ↔ L ILF	L SLF ↔ L IFOF	-	L IFOF ↔ L ATR	
L STR ↔ L CST	L ILF ↔ L IFOF	R IFOF ↔ L ATR	L UNC ↔ L STR	-	R IFOF ↔ R ATR	
R STR ↔ L CST	L SLF ↔ L PTR	R ILF ↔ L IFOF	L SLF ↔ L PTR	-	R PTR ↔ L IFOF	
L STR ↔ L AR	R STR ↔ R ATR	R PTR ↔ L IFOF	L UNC ↔ L ATR	-	L STR ↔ R SLF	
L SLF ↔ L PTR	R IFOF ↔ F MAJ	-	-	-	L PTR ↔ R ILF	
R ILF ↔ F MIN	-	-	-	-	L PTR ↔ R IFOF	
R IFOF ↔ L ATR	-	-	-	-		
L UNC ↔ L ILF	-	-	-	-		

Note. L = left hemisphere, R = right hemisphere. AR: acoustic radiation, ATR: anterior thalamic radiation, IFOF: inferior fronto-occipital fasciculus, ILF: inferior longitudinal fasciculus, MCP: middle cerebellar peduncle, PH: parahippocampal portion, PTR: posterior thalamic radiation, SLF: superior longitudinal fasciculus, STR: superior thalamic radiation, FA: fractional anisotropy, MD: mean diffusivity, ICVF: intra-cellular volume fraction, ISOVF: isotropic volume fraction, OD: orientation dispersion, λax = axial diffusivity, λrad = radial diffusivity.

Supplementary Table 10. Regression of the latent (general) factor from each of the five white matter microstructural measures on age and age^2.

**Latent factor**	**Intercept**			**Age**			**Age^2**		
	β	**SE**	***p***	***β***	**SE**	***p***	***β***	**SE**	***p***
*g*FA	0.000	0.016	0.999	−0.254	0.016	<0.001	-		-
*g*MD	−0.071	0.022	0.001	0.368	0.016	<0.001	0.071	0.015	<0.001
*g*ICVF	0.050	0.023	0.026	−0.265	0.017	<0.001	−0.051	0.016	0.001
*g*ISOVF	−0.046	0.023	0.044	0.273	0.016	<0.001	0.046	0.016	0.004
*g*OD	0.068	0.023	0.004	−0.120	0.017	<0.001	−0.068	0.017	<0.001
*g*λax	−0.088	0.022	<.001	0.341	0.016	<0.001	0.088	0.016	<0.001
*g*λrad	−0.065	0.015	0.003	0.363	0.016	<0.001	0.065	0.015	<0.001

Note. The values below correspond to the trajectories shown in Figure 6 (left panel). A model regressing the latent FA factor on age (Akaike Information Criterion = 9740.60; Bayesian Information Criterion = 9765.25) had fit that was not significantly different from a model that included both age and age^2 (AIC = 9739.86; BIC = 9758.35): $F(1) = 1.26$, $p = 0.26$. For all other models, the models including age^2 provided significantly better fit (all $p < 0.004$).

Supplementary Table 11. Relative model fit indices for each of the common, independent, and common + independent age pathway models.

**Measure**	**Model**	χ^2	**df**	**AIC**	**BIC**	p_{diff}
FA	Common	3389.12	195	−85492.07	−84863.32	-
	Independent	2855.94	174	−85983.25	−85225.05	<0.0001
	Common + Independent	2898.43	181	−85954.76	−85239.71	<0.0001
MD	Common	9767.09	219	−36176.97	−35560.55	-
	Independent	8437.64	177	−37422.42	−36547.10	<0.0001
	Common + Independent	8442.18	181	−37425.87	−36575.21	0.338
ICVF	Common	8385.93	220	257503.04	258113.30	-
	Independent	7205.96	178	256407.07	257276.23	<0.0001
	Common + Independent	7219.76	186	256404.88	257224.72	0.087
ISOVF	Common	5918.98	220	222396.66	223006.92	-
	Independent	4659.73	178	221221.41	222090.57	<0.0001
	Common + Independent	4669.02	188	221210.70	222018.21	0.505
OD	Common	3949.20	225	−137318.74	−136739.31	-
	Independent	3764.84	207	−132794.00	−131936.62	<0.0001
	Common + Independent	3052.50	193	−138151.45	−137374.76	<0.0001
λax	Common	7535.40	218	15457.67	16080.26	-
	Independent	6094.01	176	14100.28	14981.77	<0.0001
	Common + Independent	6100.60	182	14094.87	14939.37	0.360
λrad	Common	7311.37	224	38354.49	38940.09	-
	Independent	6240.41	182	37367.53	38212.03	<0.0001
	Common + Independent	6253.29	194	37356.41	38126.94	0.378

Note. Degrees of freedom differ due to the residual paths being included in the models. These residual paths were identical to those included in the age-moderation models described above. Values for p_{diff} refer to the difference (χ^2 test) between the model and the model in the row above. AIC: Akaike Information Criterion, BIC: Bayesian Information Criterion. -

Supplementary Table 12. Mediation analysis of age effects on FA by ICVF and OD.

		β	***p***	**SE**
**Regressions**				
gFA				
	age	−0.060	<0.001	0.006
	gICVF	0.925	<0.001	0.006
	gOD	−0.464	<0.001	0.007
gICVF	age	−0.251	<0.001	0.016
gOD	age	−0.095	<0.001	0.015
**Covariances**				
gICVF	gOD	0.239	<0.001	0.015
**Mediation Parameters**				
gICVF	IDE	−0.232	<0.001	0.015
gOD	IDE	0.044	<0.001	0.007
sum	IDE	−0.188	<0.001	0.015
total		−0.247	<0.001	0.016

Note. Standardised betas reported, IDE = indirect effects, SE = standard error, gFA = general factor of fractional anisotropy, gICVF = general factor of intracellular volume fraction, gOD = general factor of orientation dispersion.

Supplementary Table 13. Descriptive statistics and regression models of age and sex on brain volumetric indices.

	**Mean (SD)**	**Age**	**Age^2**	**Sex**	**Sex*Age**	R^2
Total Brain Volume†	**1174150.00 (111919.80)**	**−0.579 (< 0.001)**	−0.031 (0.026)	**−0.121 (< 0.001)**	**0.101 (< 0.001)**	0.299
Grey Matter Volume†	**621076.8 (55761.55)**	**−0.615 (< 0.001)**	−0.033 (0.009)	**−0.302 (< 0.001)**	**0.117 (< 0.001)**	0.423
White Matter Volume	**553073.30 (62228.07)**	**−0.307 (< 0.001)**	-	**0.135 (< 0.001)**	0.040 (0.212)	0.094
Left Hippocampal V.†	**3827.15 (467.02)**	**−0.223 (< 0.001)**	−0.038 (0.015)	**−0.249 (< 0.001)**	-0.037 (0.251)	0.125
Right Hippocampal V.†	**3937.95 (481.12)**	**−0.204 (< 0.001)**	**−0.069 (< 0.001)**	**−0.230 (< 0.001)**	-0.063 (0.050)	0.113
Left Thalamus Volume	**7824.38 (754.81)**	**-0.399 (<0.001)**	**-**	**-0.231 (<0.001)**	-0.003 (0.834)	0.222
Right Thalamus Volume†	**7629.53 (729.26)**	**-0.415 (<0.001)**	**-0.032 (<0.001)**	**-0.221 (<0.001)**	0.001 (0.9402)	0.229

Note. Mean and SD are of raw (unscaled) volumes (mm^3), regression models are corrected for head size. Standardised *β*s (*p* values) and adjusted R^2 reported. †quadratic model significantly better fit ($p < 0.05$). Bold type indicates significant effect ($p < 0.001$). Female coded as 0. All variables corrected for head size. Hippocampal volume is the average of left and right.

Supplementary Table 14. Correlation matrix among brain diffusion factor scores and volumetric measures.

	**gFA**	**gMD**	**gICVF**	**gISOVF**	**gOD**	gλax	gλrad	**TBV**	**GMV**	**WMV**	**lHCV**	**rHCV**	LThalV	RThalV
*g*FA														
*g*MD	-0.782													
*g*ICVF	0.833	-0.871												
*g*ISOVF	-0.039†	0.334	0.149											
*g*OD	0.158	0.386	-0.289	0.133										
gλax	-0.486	0.901	-0.715	0.402	0.682									
gλrad	-0.886	0.973	-0.889	0.276	0.205	0.800								
TBV	0.166	-0.276	0.222	-0.182	-0.083	-0.251	-0.269							
GM	0.201	-0.311	0.238	-0.229	-0.152	-0.279	-0.305	0.847						
WM	0.062	-0.128	0.117	-0.058	0.029†	-0.121	-0.123	0.789	0.341					
lHCV	0.022†	-0.169	0.106	-0.153	-0.152	-0.176	-0.145	0.374	0.410	0.189				
rHCV	0.031†	-0.171	0.103	-0.161	-0.132	-0.175	-0.147	0.376	0.390	0.214	0.620			
LThalV	0.081	-0.246	0.193	-0.155	-0.154	-0.242	-0.226	0.645	0.591	0.458	0.541	0.476		
RThalV	0.091	-0.262	0.199	-0.173	-0.162	-0.259	-0.241	0.660	0.609	0.463	0.496	0.539	0.916	
Age	-0.254	0.351	-0.252	0.263	0.104	0.320	0.347	-0.531	-0.573	-0.277	-0.250	-0.234	-0.411	-0.424

Note. Pearson's *r* reported. †not significant at $p < 0.001$. Volumetric measures are corrected for head size.

Supplementary Table 15. Regression models in training and test halves of the full sample following elastic net regression results for MRI predictors of age variance.

	Training Set (n = 1756)		***Test Set (n = 1757)***	
	β	***P***	***β***	***P***
gFA	-0.085	0.012	-0.071	0.035
gMD	0.085	0.019	0.109	0.003
gISOVF	0.119	< 0.001	0.124	< 0.001
Total Brain Volume	-0.160	< 0.001	-0.171	< 0.001
Grey Matter Volume	-0.375	< 0.001	-0.348	< 0.001

Note. Standardised *β*s and *p* values reported. Adjusted R^2 for Training Set = 0.376 and Test Set = 0.377. Training and Test sets did not differ significantly by mean age (t (3510.8) = -0.803, p = 0.422) or age variance (F (1755) = 0.983, p = 0.723). Variance inflation factors all < 3.84.

Supplementary Table 16. Regression models for thalamic radiation microstructure and thalamic volume as predictors of age variance beyond general brain atrophy.

	Loadings (%var)	*g*TR	Thalamus	TBV	R^2	VIF
FA	**≥0.794 (67.5%)**	**-0.141 (< 0.001)**	**-0.300 (< 0.001)**	**-**	0.129	≤1.05
		**-0.120 (< 0.001)**	**-0.153 (< 0.001)**	**-0.468 (< 0.001)**	0.324	≤1.16
MD	**≥0.856 (78.5%)**	**0.403 (< 0.001)**	**-0.248 (< 0.001)**	**-**	0.265	≤1.05
		**0.212 (< 0.001)**	**-0.151 (< 0.001)**	**-0.378 (<0.001)**	0.379	≤1.25
ICVF	**≥0.907 (86.7%)**	**-0.258 (< 0.001)**	**-0.291 (< 0.001)**	**-**	0.175	≤1.03
		**-0.161 (< 0.001)**	**-0.165 (< 0.001)**	**-0.435 (< 0.001)**	0.335	≤1.18
ISOVF	**≥0.768 (67.0%)**	**0.333 (< 0.001)**	**-0.280 (< 0.001)**	**-**	0.218	≤1.03
		**0.229 (< 0.001)**	**-0.163 (< 0.001)**	**0.410 (< 0.001)**	0.358	≤1.20
OD	**≥0.666 (54.1%)**	**-0.342 (< 0.001)**	**-0.392 (< 0.001)**	**-**	0.224	≤1.03
		**-0.227 (< 0.001)**	**-0.242 (< 0.001)**	**-0.403 (< 0.001)**	0.356	≤1.22

Note. Standardised βs (p values) and adjusted R^2 reported. Loadings (%var) = loadings and percentage of variance explained by the first unrotated solution of a principal component analysis of the three thalamic radiations, *g*TR = general measure of thalamic radiation microstructure, TBV = total brain volume. Bold type indicates significant effect ($p < 0.001$). Volumes corrected for head size.

Supplementary Table 17. Statistical significance tests for λ_1 and λ_1' parameters (main effects and age moderation effects of general factors) in the dedifferentiation models for FA, MD, ICVF, ISOVF and OD (Equation S2 specification).

**Tract**	**Parameter**	**FA**			**MD**			**ICVF**			**ISOVF**			**OD**		
		**Est.**	**SE**	***p***	**Est.**	**SE**	***p***	**Est.**	**SE**	***p***	**Est.**	**SE**	***p***	**Est.**	**SE**	***p***
Left AR	λ_1	**1.271**	**0.088**	**<0.001**	**0.164**	**0.016**	**<0.001**	**2.152**	**0.092**	**<0.001**	**0.802**	**0.094**	**<0.001**	**0.542**	**0.073**	**<0.001**
	λ_1'	0.024	0.046	0.607	0.089	0.082	0.276	**0.103**	**0.049**	**0.036**	-0.039	0.048	0.416	-0.032	0.038	0.398
Right AR	λ_1	**1.252**	**0.082**	**<0.001**	**0.163**	**0.014**	**<0.001**	**2.024**	**0.090**	**<0.001**	**0.727**	**0.081**	**<0.001**	**0.685**	**0.068**	**<0.001**
	λ_1'	0.017	0.043	0.692	**0.199**	**0.075**	**0.008**	**0.154**	**0.049**	**0.002**	0.043	0.042	0.306	**-0.122**	**0.036**	**0.001**
Left ATR	λ_1	**1.100**	**0.061**	**<0.001**	**0.122**	**0.009**	**<0.001**	**1.944**	**0.086**	**<0.001**	**0.375**	**0.041**	**<0.001**	**0.730**	**0.043**	**<0.001**
	λ_1'	**0.148**	**0.033**	**<0.001**	**0.621**	**0.050**	**<0.001**	**0.257**	**0.047**	**<0.001**	**0.166**	**0.022**	**<0.001**	-0.008	0.022	0.728
Right ATR	λ_1	**1.041**	**0.060**	**<0.001**	**0.127**	**0.009**	**<0.001**	**1.940**	**0.086**	**<0.001**	**0.426**	**0.039**	**<0.001**	**0.788**	**0.044**	**<0.001**
	λ_1'	**0.143**	**0.032**	**<0.001**	**0.602**	**0.049**	**<0.001**	**0.249**	**0.047**	**<0.001**	**0.161**	**0.022**	**<0.001**	-0.011	0.023	0.623
Left CingG	λ_1	**1.470**	**0.134**	**<0.001**	**0.186**	**0.010**	**<0.001**	**2.383**	**0.123**	**<0.001**	**0.767**	**0.060**	**<0.001**	**0.536**	**0.075**	**<0.001**
	λ_1'	0.026	0.071	0.711	0.035	0.051	0.493	0.076	0.065	0.247	-0.023	0.032	0.467	0.015	0.041	0.715
Right CingG	λ_1	**1.163**	**0.137**	**<0.001**	**0.182**	**0.010**	**<0.001**	**2.203**	**0.117**	**<0.001**	**0.874**	**0.056**	**<0.001**	**0.504**	**0.086**	**<0.001**
	λ_1'	0.112	0.072	0.118	0.057	0.051	0.262	0.092	0.062	0.139	**-0.060**	**0.029**	**0.039**	0.041	0.046	0.371
Left CST	λ_1	**1.009**	**0.090**	**<0.001**	**0.111**	**0.009**	**<0.001**	**1.544**	**0.080**	**<0.001**	**0.390**	**0.057**	**<0.001**	**0.331**	**0.061**	**<0.001**
	λ_1'	0.026	0.047	0.582	**0.154**	**0.046**	**0.001**	**0.129**	**0.043**	**0.003**	0.047	0.030	0.118	-0.045	0.032	0.160
Right CST	λ_1	**1.067**	**0.092**	**<0.001**	**0.124**	**0.009**	**<0.001**	**1.492**	**0.081**	**<0.001**	**0.523**	**0.055**	**<0.001**	**0.337**	**0.059**	**<0.001**
	λ_1'	-0.001	0.048	0.986	**0.115**	**0.046**	**0.013**	**0.146**	**0.043**	**0.001**	0.032	0.029	0.267	-0.031	0.031	0.308
Forceps Major	λ_1	**1.195**	**0.103**	**<0.001**	**0.163**	**0.020**	**<0.001**	**2.130**	**0.102**	**<0.001**	**0.440**	**0.111**	**<0.001**	**0.346**	**0.044**	**<0.001**
	λ_1'	**0.141**	**0.055**	**0.011**	**0.273**	**0.107**	**0.011**	**0.151**	**0.056**	**0.007**	**0.160**	**0.058**	**0.006**	-0.008	0.023	0.718
Forceps Minor	λ_1	**1.357**	**0.069**	**<0.001**	**0.201**	**0.011**	**<0.001**	**2.910**	**0.111**	**<0.001**	**0.954**	**0.066**	**<0.001**	**0.588**	**0.042**	**<0.001**
	λ_1'	**0.160**	**0.037**	**<0.001**	**0.310**	**0.061**	**<0.001**	**0.129**	**0.059**	**0.029**	0.032	0.035	0.364	-0.010	0.022	0.649
Left IFOF	λ_1	**1.507**	**0.069**	**<0.001**	**0.171**	**0.009**	**<0.001**	**2.601**	**0.094**	**<0.001**	**0.618**	**0.037**	**<0.001**	**0.698**	**0.040**	**<0.001**
	λ_1'	**0.153**	**0.037**	**<0.001**	**0.435**	**0.049**	**<0.001**	**0.207**	**0.051**	**<0.001**	**0.151**	**0.020**	**<0.001**	0.005	0.021	0.826
Right IFOF	λ_1	**1.366**	**0.066**	**<0.001**	**0.203**	**0.009**	**<0.001**	**2.760**	**0.098**	**<0.001**	**0.728**	**0.038**	**<0.001**	**0.681**	**0.038**	**<0.001**
	λ_1'	**0.165**	**0.036**	**<0.001**	**0.327**	**0.049**	**<0.001**	**0.180**	**0.053**	**0.001**	**0.146**	**0.021**	**<0.001**	0.004	0.020	0.843
Left ILF	λ_1	**1.315**	**0.067**	**<0.001**	**0.167**	**0.010**	**<0.001**	**2.452**	**0.092**	**<0.001**	**0.582**	**0.040**	**<0.001**	**0.524**	**0.033**	**<0.001**
	λ_1'	**0.132**	**0.036**	**<0.001**	**0.416**	**0.054**	**<0.001**	**0.210**	**0.050**	**<0.001**	**0.177**	**0.022**	**<0.001**	0.006	0.018	0.728
Right ILF	λ_1	**1.317**	**0.062**	**<0.001**	**0.195**	**0.009**	**<0.001**	**2.556**	**0.093**	**<0.001**	**0.766**	**0.039**	**<0.001**	**0.458**	**0.032**	**<0.001**
	λ_1'	**0.130**	**0.033**	**<0.001**	**0.319**	**0.051**	**<0.001**	**0.181**	**0.050**	**<0.001**	**0.140**	**0.022**	**<0.001**	0.030	0.017	0.070
Left PTR	λ_1	**0.910**	**0.076**	**<0.001**	**0.155**	**0.013**	**<0.001**	**2.074**	**0.093**	**<0.001**	**0.756**	**0.076**	**<0.001**	**0.375**	**0.041**	**<0.001**
	λ_1'	**0.160**	**0.041**	**<0.001**	**0.475**	**0.071**	**<0.001**	**0.221**	**0.050**	**<0.001**	**0.132**	**0.042**	**0.001**	**0.043**	**0.021**	**0.044**
Right PTR	λ_1	**0.927**	**0.076**	**<0.001**	**0.180**	**0.012**	**<0.001**	**2.214**	**0.096**	**<0.001**	**0.866**	**0.073**	**<0.001**	**0.339**	**0.041**	**<0.001**
	λ_1'	**0.142**	**0.041**	**<0.001**	**0.389**	**0.067**	**<0.001**	**0.197**	**0.052**	**<0.001**	**0.126**	**0.040**	**0.002**	**0.059**	**0.022**	**0.007**
Left SLF	λ_1	**1.391**	**0.071**	**<0.001**	**0.183**	**0.008**	**<0.001**	**2.633**	**0.102**	**<0.001**	**0.684**	**0.044**	**<0.001**	**0.563**	**0.050**	**<0.001**
	λ_1'	**0.168**	**0.038**	**<0.001**	**0.404**	**0.046**	**<0.001**	**0.248**	**0.055**	**<0.001**	**0.160**	**0.024**	**<0.001**	-0.011	0.026	0.685
Right SLF	λ_1	**1.343**	**0.067**	**<0.001**	**0.186**	**0.008**	**<0.001**	**2.576**	**0.103**	**<0.001**	**0.844**	**0.046**	**<0.001**	**0.624**	**0.051**	**<0.001**
	λ_1'	**0.168**	**0.036**	**<0.001**	**0.396**	**0.047**	**<0.001**	**0.260**	**0.056**	**<0.001**	**0.123**	**0.025**	**<0.001**	-0.019	0.027	0.484
Left STR	λ_1	**0.712**	**0.067**	**<0.001**	**0.136**	**0.008**	**<0.001**	**1.661**	**0.082**	**<0.001**	**0.593**	**0.044**	**<0.001**	**0.385**	**0.061**	**<0.001**
	λ_1'	**0.199**	**0.036**	**<0.001**	**0.366**	**0.046**	**<0.001**	**0.269**	**0.045**	**<0.001**	**0.110**	**0.024**	**<0.001**	0.008	0.033	0.799
Right STR	λ_1	**0.700**	**0.071**	**<0.001**	**0.153**	**0.009**	**<0.001**	**1.699**	**0.084**	**<0.001**	**0.648**	**0.047**	**<0.001**	**0.423**	**0.063**	**<0.001**
	λ_1'	**0.189**	**0.038**	**<0.001**	**0.288**	**0.048**	**<0.001**	**0.244**	**0.046**	**<0.001**	**0.096**	**0.025**	**<0.001**	-0.004	0.033	0.906
Left Unc	λ_1	**1.456**	**0.090**	**<0.001**	**0.146**	**0.014**	**<0.001**	**2.027**	**0.101**	**<0.001**	**0.253**	**0.042**	**<0.001**	**0.677**	**0.069**	**<0.001**
	λ_1'	0.018	0.048	0.704	**0.262**	**0.071**	**<0.001**	0.075	0.053	0.159	**0.122**	**0.023**	**<0.001**	-0.003	0.036	0.939
Right Unc	λ_1	**1.166**	**0.076**	**<0.001**	**0.154**	**0.011**	**<0.001**	**1.961**	**0.089**	**<0.001**	**0.408**	**0.037**	**<0.001**	**0.628**	**0.063**	**<0.001**
	λ_1'	0.062	0.040	0.124	**0.256**	**0.058**	**<0.001**	0.094	0.048	0.050	**0.097**	**0.020**	**<0.001**	0.021	0.034	0.525

Note. All estimates are unstandardized. Parameters that were statistically significantly different from zero are in bold. These figures correspond to the trends shown in the leftmost panels of Figures S8-S12.

Supplementary Table 18. Statistical significance tests for λ_1 and λ_1' parameters (main effects and age moderation effects of general factors) in the dedifferentiation models for λax and λrad (Equation S2 specification).

**Tract**	**Parameter**	**λax**			**λrad**		
		**Est.**	**SE**	***p***	**Est.**	**SE**	***p***
Left AR	λ_1	**0.185**	**0.020**	**<0.001**	**0.269**	**0.023**	**<0.001**
	λ_1'	0.006	0.010	0.545	0.014	0.012	0.258
Right AR	λ_1	**0.186**	**0.017**	**<0.001**	**0.259**	**0.022**	**<0.001**
	λ_1'	0.013	0.009	0.138	**0.028**	**0.011**	**0.013**
Left ATR	λ_1	**0.175**	**0.010**	**<0.001**	**0.197**	**0.014**	**<0.001**
	λ_1'	**0.045**	**0.006**	**<0.001**	**0.089**	**0.008**	**<0.001**
Right ATR	λ_1	**0.172**	**0.010**	**<0.001**	**0.190**	**0.014**	**<0.001**
	λ_1'	**0.047**	**0.006**	**<0.001**	**0.089**	**0.008**	**<0.001**
Left CingG	λ_1	**0.272**	**0.022**	**<0.001**	**0.272**	**0.018**	**<0.001**
	λ_1'	-0.019	0.012	0.111	0.018	0.010	0.067
Right	λ_1	**0.254**	**0.021**	**<0.001**	**0.254**	**0.018**	**<0.001**
CingG	λ_1'	-0.012	0.011	0.274	**0.021**	**0.010**	**0.031**
Left CST	λ_1	**0.142**	**0.015**	**<0.001**	**0.181**	**0.015**	**<0.001**
	λ_1'	0.013	0.008	0.102	**0.022**	**0.008**	**0.006**
Right CST	λ_1	**0.148**	**0.015**	**<0.001**	0.198	0.015	**<0.001**
	λ_1'	0.013	0.008	0.097	0.013	0.008	0.098
Forceps	λ_1	**0.221**	**0.024**	**<0.001**	**0.267**	**0.032**	**<0.001**
Major	λ_1'	-0.003	0.013	0.823	**0.057**	**0.017**	**0.001**
Forceps	λ_1	**0.262**	**0.015**	**<0.001**	**0.290**	**0.018**	**<0.001**
Minor	λ_1'	0.007	0.008	0.353	**0.055**	**0.010**	**<0.001**
Left IFOF	λ_1	**0.213**	**0.012**	**<0.001**	**0.303**	**0.014**	**<0.001**
	λ_1'	**0.030**	**0.006**	**<0.001**	**0.063**	**0.008**	**<0.001**
Right IFOF	λ_1	**0.238**	**0.012**	**<0.001**	**0.324**	**0.014**	**<0.001**
	λ_1'	**0.019**	**0.006**	**0.002**	**0.056**	**0.007**	**<0.001**
Left ILF	λ_1	**0.189**	**0.012**	**<0.001**	**0.299**	**0.016**	**<0.001**
	λ_1'	**0.032**	**0.007**	**<0.001**	**0.062**	**0.008**	**<0.001**
Right ILF	λ_1	**0.200**	**0.011**	**<0.001**	**0.321**	**0.014**	**<0.001**
	λ_1'	**0.027**	**0.006**	**<0.001**	**0.052**	**0.008**	**<0.001**
Left PTR	λ_1	**0.173**	**0.016**	**<0.001**	**0.230**	**0.020**	**<0.001**
	λ_1'	**0.045**	**0.009**	**<0.001**	**0.073**	**0.011**	**<0.001**
Right PTR	λ_1	**0.188**	**0.015**	**<0.001**	**0.272**	**0.020**	**<0.001**
	λ_1'	**0.045**	**0.008**	**<0.001**	**0.057**	**0.011**	**<0.001**
Left SLF	λ_1	**0.198**	**0.010**	**<0.001**	**0.283**	**0.014**	**<0.001**
	λ_1'	**0.033**	**0.005**	**<0.001**	**0.063**	**0.008**	**<0.001**
Right SLF	λ_1	**0.207**	**0.010**	**<0.001**	**0.284**	**0.014**	**<0.001**
	λ_1'	**0.031**	**0.006**	**<0.001**	**0.061**	**0.008**	**<0.001**
Left STR	λ_1	**0.163**	**0.013**	**<0.001**	**0.174**	**0.013**	**<0.001**
	λ_1'	**0.038**	**0.007**	**<0.001**	**0.058**	**0.007**	**<0.001**
Right STR	λ_1	**0.184**	**0.013**	**<0.001**	**0.192**	**0.014**	**<0.001**
	λ_1'	**0.029**	**0.007**	**<0.001**	**0.048**	**0.008**	**<0.001**
Left Unc	λ_1	**0.144**	**0.016**	**<0.001**	**0.303**	**0.022**	**<0.001**
	λ_1'	**0.021**	**0.008**	**0.009**	**0.029**	**0.012**	**0.011**
Right Unc	λ_1	**0.160**	**0.013**	**<0.001**	**0.277**	**0.017**	**<0.001**
	λ_1'	**0.020**	**0.007**	**0.003**	**0.034**	**0.009**	**<0.001**

Note. All estimates are unstandardized. Parameters that were statistically significantly different from zero are in bold. These figures correspond to the trends shown in the leftmost panels of Figures S8-S12.

Supplementary Table 19. Statistical significance tests for λ_2 and λ_2' parameters (main effects and age moderation effects of tract-specific uniquenesses) in the dedifferentiation models for FA, MD, ICVF, ISOVF and OD (Equation S2 specification).

Tract	Parameter	FA			MD			ICVF			ISOVF			OD		
		Est.	SE	*p*	Est.	SE	*p*	Est.	SE	*p*	Est.	SE	*p*	Est.	SE	*p*
Left AR	λ_2	**1.749**	**0.048**	**<0.001**	**0.345**	**0.010**	**<0.001**	**1.381**	**0.036**	**<0.001**	**2.099**	**0.057**	**<0.001**	**1.553**	**0.046**	**<0.001**
	λ_2'	-0.014	0.024	0.565	-0.049	0.049	0.315	0.004	0.018	0.842	**-0.080**	**0.029**	**0.006**	-0.006	0.024	0.804
Right AR	λ_2	**1.574**	**0.045**	**<0.001**	**0.316**	**0.009**	**<0.001**	**1.331**	**0.035**	**<0.001**	**1.782**	**0.051**	**<0.001**	**1.417**	**0.042**	**<0.001**
	λ_2'	0.024	0.023	0.293	**-0.096**	**0.045**	**0.034**	**0.034**	**0.017**	**0.047**	-0.047	0.026	0.076	0.006	0.021	0.794
Left ATR	λ_2	**1.070**	**0.026**	**<0.001**	**0.154**	**0.004**	**<0.001**	**1.186**	**0.026**	**<0.001**	**0.813**	**0.018**	**<0.001**	**0.819**	**0.019**	**<0.001**
	λ_2'	0.009	0.012	0.467	**0.095**	**0.016**	**<0.001**	**0.029**	**0.011**	**0.009**	**0.035**	**0.008**	**<0.001**	-0.012	0.008	0.146
Right ATR	λ_2	**1.033**	**0.024**	**<0.001**	**0.147**	**0.003**	**<0.001**	**1.184**	**0.025**	**<0.001**	**0.742**	**0.018**	**<0.001**	**0.789**	**0.020**	**<0.001**
	λ_2'	**0.035**	**0.011**	**0.002**	**0.128**	**0.015**	**<0.001**	**0.047**	**0.011**	**<0.001**	**0.054**	**0.008**	**<0.001**	**0.020**	**0.009**	**0.031**
Left CingG	λ_2	**2.743**	**0.073**	**<0.001**	**0.175**	**0.004**	**<0.001**	**2.243**	**0.054**	**<0.001**	**1.206**	**0.034**	**<0.001**	**1.496**	**0.044**	**<0.001**
	λ_2'	**0.078**	**0.037**	**0.034**	**0.065**	**0.020**	**0.001**	-0.004	0.026	0.864	**0.053**	**0.017**	**0.002**	**0.086**	**0.023**	**<0.001**
Right CingG	λ_2	**2.935**	**0.076**	**<0.001**	**0.175**	**0.004**	**<0.001**	**2.123**	**0.051**	**<0.001**	**1.123**	**0.030**	**<0.001**	**1.787**	**0.049**	**<0.001**
	λ_2'	-0.003	0.038	0.935	**0.057**	**0.020**	**0.003**	-0.006	0.024	0.808	-0.001	0.014	0.924	0.032	0.025	0.196
Left CST	λ_2	**1.837**	**0.036**	**<0.001**	**0.182**	**0.004**	**<0.001**	**1.378**	**0.026**	**<0.001**	**1.228**	**0.029**	**<0.001**	**1.322**	**0.023**	**<0.001**
	λ_2'	0.012	0.016	0.444	0.028	0.022	0.205	**0.025**	**0.011**	**0.026**	0.013	0.014	0.360	-0.012	0.010	0.205
Right CST	λ_2	**1.901**	**0.037**	**<0.001**	**0.180**	**0.004**	**<0.001**	**1.484**	**0.026**	**<0.001**	**1.124**	**0.027**	**<0.001**	**1.280**	**0.023**	**<0.001**
	λ_2'	-0.003	0.017	0.867	0.020	0.021	0.337	-0.005	0.011	0.691	**0.041**	**0.014**	**0.002**	-0.016	0.010	0.104
Forceps Major	λ_2	**1.967**	**0.063**	**<0.001**	**0.428**	**0.013**	**<0.001**	**1.573**	**0.052**	**<0.001**	**2.384**	**0.070**	**<0.001**	**0.913**	**0.029**	**<0.001**
	λ_2'	**0.111**	**0.034**	**0.001**	**0.163**	**0.068**	**0.017**	**0.131**	**0.029**	**<0.001**	0.032	0.036	0.378	0.015	0.015	0.328
Forceps Minor	λ_2	**1.139**	**0.040**	**<0.001**	**0.199**	**0.006**	**<0.001**	**1.277**	**0.040**	**<0.001**	**1.258**	**0.040**	**<0.001**	**0.809**	**0.026**	**<0.001**
	λ_2'	-0.002	0.021	0.909	**0.089**	**0.034**	**0.009**	**0.055**	**0.021**	**0.008**	**0.046**	**0.021**	**0.028**	0.018	0.014	0.182
Left IFOF	λ_2	**1.068**	**0.027**	**<0.001**	**0.139**	**0.003**	**<0.001**	**0.800**	**0.016**	**<0.001**	**0.636**	**0.015**	**<0.001**	**0.724**	**0.023**	**<0.001**
	λ_2'	0.005	0.012	0.710	**0.059**	**0.010**	**<0.001**	**0.035**	**0.007**	**<0.001**	**0.018**	**0.007**	**0.007**	-0.001	0.011	0.955
Right IFOF	λ_2	**1.010**	**0.023**	**<0.001**	**0.125**	**0.003**	**<0.001**	**0.868**	**0.021**	**<0.001**	**0.596**	**0.017**	**<0.001**	**0.675**	**0.022**	**<0.001**
	λ_2'	0.012	0.010	0.222	**0.056**	**0.013**	**<0.001**	-0.001	0.009	0.935	**0.021**	**0.008**	**0.010**	0.006	0.011	0.553
Left ILF	λ_2	**1.133**	**0.022**	**<0.001**	**0.174**	**0.003**	**<0.001**	**0.970**	**0.018**	**<0.001**	**0.724**	**0.015**	**<0.001**	**0.616**	**0.018**	**<0.001**
	λ_2'	-0.009	0.008	0.310	**0.022**	**0.009**	**0.015**	**0.012**	**0.006**	**0.047**	**0.013**	**0.006**	**0.038**	0.010	0.009	0.268
Right ILF	λ_2	**0.923**	**0.020**	**<0.001**	**0.145**	**0.003**	**<0.001**	**0.904**	**0.016**	**<0.001**	**0.597**	**0.017**	**<0.001**	**0.600**	**0.015**	**<0.001**
	λ_2'	0.009	0.008	0.226	**0.058**	**0.011**	**<0.001**	**0.016**	**0.006**	**0.008**	**0.025**	**0.007**	**0.001**	-0.001	0.008	0.922
Left PTR	λ_2	**1.510**	**0.028**	**<0.001**	**0.255**	**0.006**	**<0.001**	**1.382**	**0.025**	**<0.001**	**1.516**	**0.033**	**<0.001**	**0.836**	**0.022**	**<0.001**
	λ_2'	**0.027**	**0.012**	**0.022**	**0.130**	**0.025**	**<0.001**	0.012	0.009	0.205	**0.089**	**0.014**	**<0.001**	-0.007	0.011	0.537
Right PTR	λ_2	**1.500**	**0.029**	**<0.001**	**0.221**	**0.005**	**<0.001**	**1.377**	**0.025**	**<0.001**	**1.386**	**0.030**	**<0.001**	**0.835**	**0.022**	**<0.001**
	λ_2'	0.017	0.013	0.177	**0.195**	**0.024**	**<0.001**	**0.032**	**0.010**	**0.001**	**0.102**	**0.014**	**<0.001**	0.013	0.011	0.246
Left SLF	λ_2	**1.160**	**0.029**	**<0.001**	**0.110**	**0.003**	**<0.001**	**1.120**	**0.026**	**<0.001**	**0.797**	**0.016**	**<0.001**	**1.014**	**0.022**	**<0.001**
	λ_2'	-0.005	0.013	0.705	**0.060**	**0.014**	**<0.001**	**0.037**	**0.012**	**0.002**	**0.019**	**0.006**	**0.002**	0.003	0.010	0.769
Right SLF	λ_2	**1.105**	**0.029**	**<0.001**	**0.117**	**0.003**	**<0.001**	**1.269**	**0.028**	**<0.001**	**0.760**	**0.017**	**<0.001**	**1.032**	**0.022**	**<0.001**
	λ_2'	-0.015	0.014	0.267	**0.064**	**0.015**	**<0.001**	-0.003	0.012	0.809	**0.031**	**0.007**	**<0.001**	-0.008	0.010	0.411
Left STR	λ_2	**1.349**	**0.024**	**<0.001**	**0.155**	**0.003**	**<0.001**	**1.310**	**0.025**	**<0.001**	**0.865**	**0.017**	**<0.001**	**1.279**	**0.021**	**<0.001**
	λ_2'	**0.021**	**0.009**	**0.021**	**0.029**	**0.014**	**0.044**	0.020	0.010	0.053	**0.013**	**0.006**	**0.041**	**0.029**	**0.008**	**<0.001**
Right STR	λ_2	**1.457**	**0.025**	**<0.001**	**0.164**	**0.003**	**<0.001**	**1.397**	**0.024**	**<0.001**	**0.926**	**0.017**	**<0.001**	**1.337**	**0.021**	**<0.001**
	λ_2'	0.004	0.009	0.687	**0.031**	**0.014**	**0.020**	0.008	0.009	0.407	0.010	0.007	0.140	-0.004	0.007	0.628
Left Unc	λ_2	**1.709**	**0.048**	**<0.001**	**0.289**	**0.006**	**<0.001**	**1.815**	**0.041**	**<0.001**	**0.860**	**0.023**	**<0.001**	**1.402**	**0.039**	**<0.001**
	λ_2'	0.023	0.025	0.359	-0.016	0.025	0.524	**-0.044**	**0.019**	**0.019**	**0.044**	**0.012**	**<0.001**	0.013	0.020	0.513
Right Unc	λ_2	**1.438**	**0.043**	**<0.001**	**0.210**	**0.005**	**<0.001**	**1.388**	**0.034**	**<0.001**	**0.703**	**0.021**	**<0.001**	**1.229**	**0.036**	**<0.001**
	λ_2'	0.029	0.022	0.194	**0.075**	**0.023**	**0.001**	**0.041**	**0.016**	**0.011**	**0.057**	**0.011**	**<0.001**	**0.053**	**0.019**	**0.005**

Note. All estimates unstandardized. Parameters that were statistically significantly different from zero are in bold. These figures correspond to the age trends shown in the centre panels of Figures S10-16.

Supplementary Table 20. Statistical significance tests for λ_2 and λ_2' parameters (main effects and age moderation effects of tract-specific uniquenesses) in the dedifferentiation models for λax and λrad (Equation S2 specification).

Tract	Parameter	λax			λrad		
		Est.	SE	*p*	Est.	SE	*p*
Left	λ_2	**0.447**	**0.013**	**<0.001**	**0.494**	**0.014**	**<0.001**
AR	λ_2'	-0.006	0.006	0.373	-0.005	0.007	0.492
Right	λ_2	**0.377**	**0.011**	**<0.001**	**0.471**	**0.013**	**<0.001**
AR	λ_2'	-0.004	0.006	0.461	-0.012	0.007	0.064
Left	λ_2	**0.183**	**0.004**	**<0.001**	**0.263**	**0.006**	**<0.001**
ATR	λ_2'	**0.007**	**0.002**	**<0.001**	**0.011**	**0.003**	**<0.001**
Right	λ_2	**0.180**	**0.004**	**<0.001**	**0.237**	**0.006**	**<0.001**
ATR	λ_2'	**0.011**	**0.002**	**<0.001**	**0.022**	**0.002**	**<0.001**
Left	λ_2	**0.456**	**0.012**	**<0.001**	**0.369**	**0.009**	**<0.001**
CingG	λ_2'	**0.027**	**0.006**	**<0.001**	-0.001	0.004	0.898
Right	λ_2	**0.447**	**0.012**	**<0.001**	**0.379**	**0.009**	**<0.001**
CingG	λ_2'	0.011	0.006	0.050	-0.002	0.004	0.655
Left	λ_2	**0.328**	**0.007**	**<0.001**	**0.303**	**0.007**	**<0.001**
CST	λ_2'	0.000	0.003	0.899	0.005	0.004	0.131
Right	λ_2	**0.339**	**0.007**	**<0.001**	**0.296**	**0.007**	**<0.001**
CST	λ_2'	-0.006	0.003	0.061	0.005	0.003	0.126
Forcep	λ_2	**0.524**	**0.016**	**<0.001**	**0.666**	**0.020**	**<0.001**
s Major	λ_2'	0.007	0.008	0.404	**0.027**	**0.011**	0.011
Forcep	λ_2	**0.286**	**0.010**	**<0.001**	**0.332**	**0.010**	**<0.001**
s Minor	λ_2'	0.010	0.005	0.049	0.002	0.005	0.649
Left	λ_2	**0.202**	**0.004**	**<0.001**	**0.207**	**0.005**	**<0.001**
IFOF	λ_2'	**0.006**	**0.002**	**0.007**	**0.005**	**0.002**	0.012
Right	λ_2	**0.205**	**0.006**	**<0.001**	**0.168**	**0.005**	**<0.001**
IFOF	λ_2'	0.001	0.003	0.838	**0.008**	**0.002**	0.001
Left	λ_2	**0.227**	**0.005**	**<0.001**	**0.256**	**0.006**	**<0.001**
ILF	λ_2'	0.003	0.002	0.095	0.000	0.002	0.928
Right	λ_2	**0.180**	**0.004**	**<0.001**	**0.191**	**0.004**	**<0.001**
ILF	λ_2'	**0.005**	**0.002**	**0.004**	**0.011**	**0.002**	**<0.001**
Left	λ_2	**0.320**	**0.007**	**<0.001**	**0.394**	**0.008**	**<0.001**
PTR	λ_2'	**0.015**	**0.003**	**<0.001**	**0.026**	**0.003**	**<0.001**
Right	λ_2	**0.285**	**0.007**	**<0.001**	**0.368**	**0.008**	**<0.001**
PTR	λ_2'	**0.020**	**0.003**	**<0.001**	**0.022**	**0.003**	**<0.001**
Left	λ_2	**0.170**	**0.005**	**<0.001**	**0.201**	**0.005**	**<0.001**
SLF	λ_2'	**0.004**	**0.002**	**0.024**	**0.008**	**0.002**	0.001
Right	λ_2	**0.171**	**0.004**	**<0.001**	**0.207**	**0.005**	**<0.001**
SLF	λ_2'	**0.004**	**0.002**	**0.042**	**0.008**	**0.002**	0.002
Left	λ_2	**0.248**	**0.005**	**<0.001**	**0.265**	**0.005**	**<0.001**
STR	λ_2'	**0.006**	**0.002**	**0.002**	0.004	0.002	0.124
Right	λ_2	**0.259**	**0.005**	**<0.001**	**0.272**	**0.005**	**<0.001**
STR	λ_2'	-0.001	0.002	0.465	**0.007**	**0.002**	**0.002**
Left	λ_2	**0.337**	**0.006**	**<0.001**	**0.416**	**0.010**	**<0.001**
Unc	λ_2'	0.000	0.003	0.994	**0.018**	**0.005**	**<0.001**
Right	λ_2	**0.250**	**0.005**	**<0.001**	**0.297**	**0.008**	**<0.001**
Unc	λ_2'	**0.017**	**0.002**	**<0.001**	**0.027**	**0.004**	**<0.001**

Note. All estimates are unstandardized. Parameters that were statistically significantly different from zero are in bold. These figures correspond to the age trends shown in the centre panels of Figures S10-16.
